# Report of the Vth Workshop of the Spanish National Network on Mycotoxins and Toxigenic Fungi and Their Decontamination Processes (MICOFOOD), 10–11 December 2020

**DOI:** 10.3390/toxins13010056

**Published:** 2021-01-13

**Authors:** Laura Gámiz-Gracia, Ana M. García-Campaña

**Affiliations:** Department of Analytical Chemistry, Faculty of Sciences, University of Granada, 18071 Granada, Spain; amgarcia@ugr.es

## 1. Preface

The Spanish National Network on Mycotoxins and Toxigenic Fungi and their Decontamination Processes (MICOFOOD) held its V Workshop on 10–11 December 2020. The venue was the University of Valencia, although, due to the pandemic situation, most of the participants followed the event online. Over 100 scientists, researchers, and representatives of the industry followed the Workshop, with the aim of discussing the different aspects of mycotoxin research and their impact on human and animal health, including: Study of mycotoxin-producing fungi, toxicology, analytical methods for the determination of mycotoxins, occurrence studies, reduction, and prevention, among others. 

MICOFOOD is led by Dr. Jordi Mañes Vinuesa (Laboratory of Food Chemistry and Toxicology of the University of Valencia) and includes different research groups from the universities of Extremadura, Granada, Lleida, Navarra, Santiago de Compostela, Valencia, Zaragoza, Complutense University of Madrid, Autonomous University of Barcelona, and The Spanish National Research Council (CSIC). 

Within the objectives of the Research and Innovation Framework Program “Horizon 2020”, MICOFOOD aims to promote the relationship between researchers, the food industry, and the administration in order to address and, as far as possible, minimize the problems caused by the presence of toxigenic fungi and mycotoxins in food, as well as help the implementation and improvement of quality management measures. 

The scientific program of the V Workshop of MICOFOOD included 25 oral presentations and 27 posters, covering topics such as the characterization of toxicity and toxicological studies, decontamination processes, evaluation of novel biopreservative and biocontrol agents, new developments in analytical methods for mycotoxin detection, occurrence studies in food and feed, bioaccesibility and biomonitoring studies. Two prizes were awarded:

Best Oral Presentation: B. Arce-López et al. (University of Navarra), “Biomonitoring of Mycotoxins in Plasma of Spanish Adults”.

Best Poster Presentation: M. Taroncher et al. (University of Valencia), “Toxicological Interaction between the Mycotoxin T-2 Toxin and its Modified Form in HepG2 Cells and Prediction of Toxicity by *In Silicon* Approaches”.

Finally, an interesting roundtable about the “Importance of Food Safety in the Post COVID 19 Industry” was held, with the participation of different representatives of food companies that commented the challenges that the Food Safety must afford in the near future.

## 2. Scientific Committee

Jordi Mañes Vinuesa (University of Valencia)

Giuseppe Meca (University of Valencia)

Misericordia Jiménez Escamilla (University of Valencia)

Mar Rodríguez Jovita (University of Extremadura)

Vicente Sanchis Almenar (University of Lleida)

Agustín Ariño Moneva (University of Zaragoza)

Covadonga Vázquez Estévez (Complutense University of Madrid)

Elena González-Peñas (University of Navarra)

Adela López de Cerain (University of Navarra)

Luis González Candelas (Spanish National Research Council, CSIC)

Alberto Cepeda Sáez (University of Santiago de Compostela)

Francisco Javier Cabañes Saenz (Autonomous University of Barcelona)

Ana María García Campaña (University of Granada)

## 3. Organizing Committee

Giuseppe Meca (University of Valencia)

Mónica Fernández (University of Valencia)

Jorge Calpe Ruano (University of Valencia)

Carlos Luz (University of Valencia)

Juan Manuel Quiles (University of Valencia)

Victor D’Opazo (University of Valencia)

## 4. Oral Presentations

### 4.1. Mycotoxins: In Silico Characterization of Genotoxicity

Alonso-JáureguiMaría^1^*FontMaría^2^,^3^CerainAdela López de^1^,^3^González-PeñasElena^2^VettorazziAriane^1^,^3^1Department of Pharmacology and Toxicology, School of Pharmacy and Nutrition, University of Navarra, 31008 Pamplona, Spain2Department of Pharmaceutical Technology and Chemistry, School of Pharmacy and Nutrition, University of Navarra, 31008 Pamplona, Spain3Idisna, Navarra Institute for Health Research, 31008 Pamplona, Spain*Correspondence: malonso.17@alumni.unav.es

The present study aimed at characterizing the genotoxicity of mycotoxins in silico by means of two predictive tools: DEREK Nexus^®^ (Lhasa Limited^®^), a knowledge-based expert system for qualitative toxicity prediction, and VEGA (vegahub.eu), a qualitative structure–activity relationship (QSAR) model platform. The mycotoxins chosen were clustered in groups: (i) Group 1 included aflatoxin B1 and sterigmatocystin that have a central scaffold in common (furo [2,3] benzofuran) are produced by the *Aspergillus* species within the same biosynthetic route; (ii) Group 2 included type A (T-2 toxin and HT-2 toxin) and type B thrichothecenes (nivalenol, deoxinivalenol and its acetylated forms of the OH located at position 3 and 15 and fusarenon-X). All are produced by *Fusarium* species and share a central scaffold (1,5-dimethylspiro[8-oxatricyclo[7.2.1.02,7]dodec-5-ene-12,2′-oxirane]) and (iii) Group 3 with mycotoxins with no common features (ochratoxin A, zearalenone, and fumonisin B1). The predictions from DEREK were classified as “positive” (certain, probable, plausible, or equivocal) or “negative” (improbable or nothing to report) for genotoxicity; while VEGA classification was “non-mutagenic,” “mutagenic,” and “suspected to be mutagenic” and was accompanied with a level of prediction reliability.

The predictions for aflatoxins and sterigmatocystin with DEREK indicated plausible chromosome damage in vitro and in vivo, plausible in vitro mutagenic potential, and equivocal in vivo mutagenicity. With VEGA, almost all QSAR models agreed on the mutagenic potential of both mycotoxins with good reliability. For group 2, the common prediction from DEREK showed a plausible chromosome aberration and mutagenic potential in vitro and in vivo. The overall VEGA conclusion for nivalenol, deoxynivalenol, and F-X was no-mutagenicity, with just one QSAR model presenting an appropriate reliable prediction. With respect to type A thrichothecenes, T-2 toxin was no mutagenic, though HT-2 toxin was mutagenic. The reliability of the predictions was moderate or low. For group 3, both predictive tools agreed on the absence of genotoxicity in all cases.

**Keywords:** mycotoxins; genotoxicity; in silico; VEGA; DEREK Nexus^®^

**Acknowledgments:** This work was supported by the Spanish Ministry of Economy, Industry and Competitivity through the project “Multiexposure and combined toxicity of mycotoxins in humans and farm animals. Toxicokinetic characterization and metabolism” (AGL2017-85732-R) (MINECO/AEI/FEDER, UE). M.A thanks the “Asociación de Amigos” (University of Navarra) and the Spanish Government for the predoctoral grants received “Ayudas para la Formación de Personal Investigador” and “Ayudas para contratos predoctorales para la formación de doctores 2018 (PRE2018-083527)”.

### 4.2. Biological Decontamination of Different Mycotoxins Using Probiotic Microorganisms

Gómez-AlbarránCarolina*PatiñoBelénVázquezCovadongaGil-SernaJéssicaDepartment of Genetics, Physiology and Microbiology, Faculty of Biology, University Complutense of Madrid, 28040 Madrid, Spain*Correspondence: caroli13@ucm.es

Aflatoxin B1 (AFB1), deoxynivalenol (DON), fumonisin (FUM), and ochratoxin A (OTA) are important mycotoxins since their presence in certain products intended for human or animal consumption is considered a threat to their health. Biological detoxification, and specifically, the use of probiotics and other QPS microorganisms, is one of the most promising strategies in reducing the concentration of these mycotoxins. In this study, different species of lactic acid bacteria (*Lactobacillus plantarum, Lactobacillus casei, Lactococcus lactis subsp lactis, Lactobacillus rhamnosus, Streptococcus thermophilus,* and *Enterococcus faecium*), bacteria of the genus *Bacillus*, and yeasts (*Hanseniaspora uvarum, Kazachstania unispora, Metschnikowia pulcherrima, Aureobasidium pullulans, Debaromyces hansenii, Pichia anomala,* and *Pichia manshurica*) were isolated from probiotic products and grapes and identified by 16S and ITS sequencing. Subsequently, we analyzed the ability of these microorganisms to detoxify aflatoxin B1, deoxynivalenol, fumonisin, and ochratoxin A extracts. Mycotoxins concentrations were determined using ELISA tests. Most of these microorganisms exhibited a high capacity to reduce the initial concentration of AFB1, OTA, and FUM with reduction values up to 87%, 81%, and 82%, respectively. *Lactobacillus casei* (81.56 %) and *H. uvarum* (79.4%) were the most effective species to reduce FUM, whereas *A. pullulans* (87.67%) and *M. pulcherrima* (80.92%) were the most relevant species in AFB1 and OTA detoxification, respectively. Conversely, DON concentration seemed to increase in the presence of most of the microorganisms tested, probably due to a test interference with DON degradation products.

In conclusion, these promising findings suggest the possible application of these microorganisms for mycotoxin decontamination in both humans and animals, as well as in food and feed, avoiding large economic losses and reducing the diseases associated with their exposure.

**Keywords:** fumonisin; aflatoxin B1; ochratoxin A; deoxynivalenol; biological detoxification

**Acknowledgments:** Work supported by Spanish Ministry of Science and Innovation (RTI 2018-097593-B-C21). C. Gómez-Albarrán is supported by a FPI fellowship by the Spanish Ministry of Science and Innovation (PRE 2019-087768).

### 4.3. Evaluation of Bufala Whey Milk Fermented by Lactic Acid Bacteria as a Bread Biopreservative Agent

LuzCarlos*D´OpazoVictorCalpeJorgeQuilesJuan ManuelMañesJordiMecaGiuseppeLaboratory of Food Chemistry and Toxicology, Faculty of Pharmacy, University of Valencia, 46100 Burjassot, Valencia, Spain*Correspondence: Carlos.Luz@uv.es

The aim of this study was to reevaluate Mozzarella di Bufala Campana whey fermentation by lactic acid bacteria (LAB) and the use of this ingredient as a bio-preservative in bread production. Whey was pasteurized and fermented by 9 selected LAB with antifungal activity for 72 h at 37 °C. Subsequently, fermented whey (BWF) was incorporated into the bread formulation, and the pH; antimicrobial metabolites, such as organic acids and volatile organic compounds (VOCs); total phenolic content; DPPH radical-scavenging activity and visual shelf-life were characterized. The highest lactic acid content was observed in the BWF by *L. plantarum* TR7 (15.0 g/L) and *L. plantarum* TR2 (12.5 g/L). In addition, an increase in VOCs such as a hexanal, benzeneacetaldehyde, benzaldehyde, and pyrazine tetramethyl was determined in bread with BWF. BWF by LAB evidenced an increase in radical scavengers, and this was reflected in a 33% rise in the DPPH-inhibitory activity of bread with BWF compared with the control. Breads in which 100% of the water was replaced with BWF by *L. plantarum* TR7 and *L. ghanensis* TR2 showed fungal growth at 20 days of storage and evidenced an improvement in the shelf-life by 2 and 15 days compared with a control containing calcium propionate at 0.3% and control bread, respectively.

**Keywords:** mozzarella di Bufala Campana whey; lactic acid bacteria; biopreservation; bread shelf-life

### 4.4. Bioaccessibility and Bioavailability of Phenolic Acids from Food Extracts Fermented with Lactic Acid Bacteria

EscriváLaura*MarucciAgneseLuzCarlosMañesJordiMecaGiuseppeManyesLaraDepartment of Preventive Medicine and Public Health, Food Sciencs, Toxicology and Forensic Medicine, Faculty of Pharmacy, University of Valencia, 46100 Burjassot, Valencia, Spain*Correspondence: laura.escriva@uv.es

Microbial fermentations with lactic acid bacteria (LAB) produce bioactive compounds, including phenolic acids, that are released during human digestion, may have antioxidant, antimicrobial, and antifungal activity against several mycotoxigenic fungi. The objective of the present study was to evaluate the bioaccessibility and bioavailability of phenolic acids from two food matrices—whey powder and yellow mustard flour—fermented with *Lactobacillus plantarum*, respectively. Fermented and non-fermented extracts were analyzed by LC-qTOF-MS (initial extracts). For bioaccessibility determination, each extract was subjected to a simulated human gastrodigestion system reproducing physiological steps (oral, gastric, and pancreatic digestion) by incubation at 37.5 °C with (i) artificial saliva containing α-amylase; pepsin (pH = 2; 2 h incubation); and (ii) pancreatin and bile salts (pH = 6.5; 2 h incubation). Intestinal digest (pH = 7.2) was analyzed by LC-qTOF-MS (digested extracts), and frozen (−80 °C), lyophilized, and resuspended in Hanks Balanced Salt Solution (HBSS). Bioavailability evaluation was performed on in vitro intestinal epithelium Caco-2 cells model cultured in Transwell plates (225,000 cells/well) until complete differentiation (day 21; verified by the neutral red assay). Four concentrations of digested extracts (0.2%, 0.4%, 0.6%, and 0.8%) were added in triplicates into the apical area, and aliquots of both apical and basolateral compartments were collected at different times (0, 1, 2, 3, 4 h) and analyzed by LC-qTOF-MS (absorbed extracts). Analysis of initial extracts showed the presence of phenolic acids including DL-3-Phenyllactic acid and Lactic acid in whey and mustard extracts; while Benzoic acid, P-Coumaric acid, and Sinapic acid were only present in mustard extracts. Preliminary results showed bioaccessibility of DL-3-Phenyllactic ranging between 2.8–6.0% and 1.2–3%, in mustard and whey extracts, respectively; being slightly higher in the case of fermented whey compared to the non-fermented extract. The analysis of collected apical and basolateral aliquots will allow the study of the human absorption process, as well as the bioavailability determination of these phenolic compounds.

**Keywords:** phenolic acids; lactic acid bacteria; bioaccessibility; bioavailability; Caco-2 cells

### 4.5. Biopreservation Potential of Lactic Acid Bacteria against Pathogenic Fungi on Red Grape

D´OpazoVictor*LuzCarlosQuilesJuan ManuelCarbonellRaquelMañesJordiMecaGiuseppeLaboratory of Food Chemistry and Toxicology, Faculty of Pharmacy, University of Valencia, 46100 Burjassot, Valencia, Spain*Correspondence: dotavic@uv.es

Nowadays, the increasing resistance of fungi to pesticides is a concerning issue to the industry. New antifungal methods are being proved, such as the use of microorganisms for biopreservation. In this study, the biopreservation potential of four strains of *L. plantarum* was tested against fungi from *Aspergillus* and *Botrytis*. To achieve this objective, a characterization of the antifungal activity of the fermented bacterial Man–Rogosa–Sharpe (MRS) medium was performed. In addition, the analysis of different compounds presents in the fermented medium. In the end, all four fermented mediums were tested as biopreservation of red grapes contaminated with *Aspergillus carbonarius, Aspergillus niger, Aspergillus ochraceus, Aspergillus tubingensis,* and *Botrytis cinerea*. The antifungal activity results showed that *L. plantarum* E3 and *L. plantarum* E4 exhibited the highest antifungal activities reaching minimum fungicidal concentrations from 6.3 to 100 g/L. The compounds’ analysis evidenced a wide pool of different antifungal molecules in the cell-free supernatant (CFS) that were not present in the non-fermented medium. Such as acid lactic, acetic acid, phenyllactic acid, dihydrocaffeic acid, benzoic acid ketones, and pyrazines. Finally, in the test performed on red grapes, all four CFS evidenced a significant reduction of 1.32 Log_10_ spores per gram of fruit in the grapes contaminated by *A. ochraceous* compared to the MRS medium control. Likewise, CFS produced by *L. plantarum* E3 evidenced a reduction of 0.92 Log10 spores per gram of fruit for the grapes contaminated by B. cinerea.

**Keywords:** lactic acid bacteria; *Botrytis*; *Aspergillus*; biopreservation

### 4.6. Total Polyphenols and Antioxidant Activity in Fermented Food Extracts after Simulated Digestion and In Vitro Absorption in Caco-2 Cells

MarucciAgnese*ManyesLaraMecaGiuseppeFontGuillerminaEscriváLauraDepartment of Preventive Medicine and Public Health, Food Sciencs, Toxicology and Forensic Medicine, Faculty of Pharmacy, University of Valencia, 46100 Burjassot, Valencia, Spain*Correspondence: agma@alumni.uv.es

Fermentation with lactic acid bacteria (LAB) is studied as a strategy to decrease fungal contamination and mycotoxins production since it originates bioactive compounds, such as polyphenols, with antimicrobial, antifungal, and antioxidant activity. The aim of the present study was to evaluate total polyphenols and antioxidant activity of mustard flour and whey extracts fermented with LAB after simulated human digestion and in vitro absorption in Caco-2 cells. Fermented and non-fermented extracts were subjected to a digestion process in different steps by enzyme addition (α-amylase, pepsin; pancreatin, bile salts), pH adjustment (pH = 2; 6.5; 7.2), and incubation (37.5 °C; 4 h). Digested extracts were lyophilized and resuspended at different concentrations (0.2, 0.4, 0.6, and 0.8%) in HBSS. Extracts were added into the apical part of the intestinal epithelium model once the intestinal barrier was completely formed (differentiation day 21). Cells were incubated for 4 h, and both apical and basolateral aliquots were collected at 1, 2, 3, and 4 h to be analyzed. Total polyphenols were determined by Folin–Ciocalteu Reagent microprocedure. Briefly, a 25 μL sample (or gallic acid as standard curve) were mixed with 125 μL diluted Folin (1/5) and 25μL NaCO_3_ (20%) after vigorous agitation. Absorbance was measured at 750 nm after 1 h incubation in darkness. Antioxidant activity was determined by the reduction of 100 μL 2,2-diphenyl-1-picrylhydrazyl (DPPH) after its reaction with 50 μL sample, 1 h incubation in darkness, and absorbance measurement at 517 nm. Preliminary results showed increased concentration of total polyphenols with dose increment in flour extracts collected at 2, 3, and 4 h, with values ranging between 0.3 and 20.8 mg/L. Whey extracts showed detectable polyphenols concentrations only at the highest doses (0.6, 0.8%) and at 3, 4 h (0.5–1.2 mg/L). Low antioxidant activity was observed (<6%) mainly at 3, 4 h. Results indicate that polyphenols and antioxidant compounds are present in small amounts in mustard flour and whey extracts after simulated human digestion and absorption.

**Keywords:** antioxidant activity; total polyphenols; lactic acid bacteria; intestinal absorption

### 4.7. Experimental Design to Assess Protective Role of Milk Fermented Whey and Carotenoids against AFB1 and OTA Induced Toxicity in Jurkat Cells

CorbellaMiriam*CimbaloAlessandraEscriváLauraFontGuillerminaManyesLaraLaboratory of Food Chemistry and Toxicology, Faculty of Pharmacy, University of Valencia, 46100 Burjassot, Valencia, Spain*Correspondence: micoror@alumni.uv.es

Aflatoxin B1 (AFB1) and Ochratoxin A (OTA) are mycotoxins produced by filamentous fungi belonging to *Aspergillus* and *Penicillium* genera. They are currently considered the most important mycotoxins in terms of food safety in both humans and animals. Over the years, the ability of bioactive compounds to prevent mycotoxins adverse effects have been investigated. For this purpose, the beneficial effect of milk fermented whey and pumpkin extract rich in carotenoids on AFB1 and OTA cytotoxicity will be evaluated in Jurkat T cells through a proteomic approach. Jurkat T cell culture exposed to: (a) AFB1, (b) OTA, (c) AFB1 and OTA, (d) fermented whey, (e) carotenoids, (f) fermented whey and carotenoids, (g) AFB1, OTA, and fermented whey (h) AFB1, OTA and carotenoids (i) AFB1, OTA, fermented whey and carotenoids and to control DMSO 0.5%. Proteins will be extracted from exposed cells by means of a lysis buffer (Urea 8M/Thiourea 2M/Tris-HCl 50 mM) and subsequently quantified by using a NeoDot nano-spectrophotometer. Afterward, a concentration of 1000 ppm protein extract will be reduced with dithiothreitol and alkylated with iodoacetamide at a concentration of 200 mM in order to disrupt polypeptide chains. Lastly, peptides will split in Lysine–Arginine bonds through tryptic digestion overnight. Three biological and two technical replicates of each sample will be analyzed with an LC system coupled with quadrupole time of flight (Q-TOF, Agilent) in a concentration of 100 µg/µL by using a C18 column during a 40 min run time at a flow rate of 0.5 mL/min. The data obtained will be processed with Spectrum Mill software, and the differentially expressed proteins will be statistically evaluated by using Mass Professional Profiler software (Agilent). Results will show significant differentially expressed proteins involved in Jurkat T cell functions and the impact which they may produce on human health.

**Keywords:** mycotoxin; proteomics; lymphoblastoid cells; Q-TOF; prevention

**Acknowledgments:** This work was supported by Spanish Ministry of Economy and Competitiveness (PID2019-108070RB-I00-ALI) and grant (GVPROMETEO2018-126).

### 4.8. Standardization of Near-Infrared Hyperspectral Imaging for Wheat Single Kernel Quantification and Discrimination According to Deoxynivalenol Level

FemeniasAntoni*RamosAntonio J.SanchisVicenteMarínSoniaApplied Mycology Unit, Food Technology Department, University of Lleida, UTPV-XaRTA, Agrotecnio, 25198 Lleida, Spain*Correspondence: antoni.femenias@udl.cat

Near-infrared hyperspectral imaging (HSI-NIR) introduces the spatial recognition ability to conventional NIR devices. This facility makes HSI-NIR suitable for single kernel spectral features recognition as the region of interest. Thus, the trouble of heterogeneous contamination of wheat batches by mycotoxins can be managed by sorting of wheat batches in grain industry arrival. Moreover, it would be an alternative as a routine analytical method to analyze samples taken from batches. Unlike wet chemistry analytic techniques, HSI-NIR is faster, environmentally-friendly, and non-destructive. The present work had two main objectives. The first one was to standardize HSI-NIR for individual kernel DON analysis to build a prediction model able to quantify the concentration of this mycotoxin. The second objective was the calibration of HSI-NIR to discriminate kernels above and below the EU legal limit (1250 μg/kg). UHPLC analysis of DON for individual kernels, previously scanned by HSI-NIR, was used as References method. The kernels were scanned in both a crease-up and crease-down position and for different image captures. The spectra were pretreated by Multiplicative Scatter Correction (MSC) and Standard Normal Variate (SNV), 1st and 2nd derivatives and normalization, and they were also evaluated by removing spectral tails. Firstly, the results showed that the best fitted predictive model was on SNV pretreated data with a performance of R^2^ 0.88 and RMSECV 4.8 mg/kg, for which 7 characteristic wavelengths were used. For the second objective, Linear Discriminant Analysis (LDA), Naïve Bayes, and K-nearest Neighbors models were classified with 98.9 and 98.4% of correctness 1st derivative and SNV spectra, respectively. The results demonstrated the ability of HSI-NIR as a starting point for DON management in wheat, and they are encouraging for future investigations in wheat single kernel quantification and sorting according to DON contamination in the grain industry.

**Keywords:** hyperspectral imaging; near infrared; deoxynivalenol; wheat single kernels; cereal sorting

**Acknowledgments:** This work was supported by the Spanish Ministry of Economy, Industry and Competitivity through the project “Cereal sorting and processing techniques, and their impact on deoxynivalenol contamination in baby foods” (AGL2017-87755-R) (MINECO/AEI/FEDER, UE).

### 4.9. Evaluation of S. xylosus and S. equorum as Biocontrol Agents against Ochratoxigenic Moulds in Dry-Cured Ham

CebriánEva*CorrilleroAidaMartínIreneCórdobaJuan JoséRodríguezMarFood Hygiene and Safety, Meat and Meat Products Research Institute, Faculty of Veterinary Science, University of Extremadura, 10003 Cáceres, Spain*Correspondence: evcebrianc@unex.es

The presence of moulds on the surface of dry-cured ham is common due to the environmental conditions reached during the ripening process. Some fungi are producers of ochratoxin A (OTA). This mycotoxin is highly toxic, thus it is necessary to apply preventive measures to reduce its presence. A promising strategy is the use of microorganisms usually found in this product, such as Cocci Gram +, catalase +. The aim of this work was to evaluate the antifungal effect of *S. xylosus* and *S. equorum* isolated from dry-cured ham on the growth of *P. nordicum* and *A. westerdijkiae* and OTA production at two different concentrations (10^3^ and 10^5^ cfu/mL) in dry-cured ham-based agar at 15 and 20 °C. The diameter of the colonies was measured daily for 21 days. OTA was extracted by QuEChERS methodology and quantified by Orbitrap Q Exactive Plus analysis. The two staphylococci isolates significantly decreased the growth of both moulds and OTA production at 15 and 20 °C. The reduction of both parameters by *S. xylosus* was directly proportional to the inoculated concentration of the bacteria. *S. xylosus* provoked a drop in OTA production of more than 99% in both *P. nordicum* and *A. westerdijkiae. S. equorum* significantly decreased the fungal growth even to the lowest inoculum concentration, although the reduction of OTA production, between 98% and 99%, was very similar to both concentrations. In conclusion, both staphylococci isolates showed a great antifungal activity against ochratoxigenic moulds commonly found on dry-cured ham. Consequently, their use as biocontrol agents could be an appropriate preventive measure to control the hazard associated with the presence of OTA in dry-cured ham.

**Keywords:** toxigenic moulds; *Staphylococcus xylosus*; *Staphylococcus equorum*; OTA; dry-cured ham

**Acknowledgments:** This work has been financed by the Spanish Ministry of Economy and Competitiveness, Government of Extremadura and FEDER (AGL2016-80209-P, PID2019-104260GB-100, GR18056).

### 4.10. Assessment of the Bioaccessibility of Aflatoxin B_1_ and Ochratoxin A from Wheat Bread by In Vitro Digestion

Pietrzak-FieckoRenata^1^LlorensPaula^2^*PolliniLuna^3^MañesJordi^2^JuanCristina^2^1Department of Commodities and Food Analysis, Faculty of Food Sciences, University of Warmia and Mazury, 10-719 Olsztyn, Poland2Laboratory of Food Chemistry and Toxicology, Faculty of Pharmacy, University of Valencia, 46100 Burjassot, Valencia, Spain3Food Science and Nutrition Section Department of Pharmaceutical Sciences, University of Perugia, 06126 Perugia, Italy*Correspondence: paullo3@alumni.uv.es

Bread has been one of the world’s most important foods and it is susceptible to the action of molds. Access of the mycotoxigenic fungi to the raw materials and the finished product should, therefore, be restricted through proper storage, conditioning of the flour, and an adequate indoor air quality system. Mycotoxins are the most important contaminants in cereals and related products in terms of economic effect and toxicity. In the evaluation of the oral bioavailability of mycotoxins, the first step is the determination of its bioaccessibility.

The present study aims to investigate the bioaccessibility of AFB_1_ and OTA from wheat bread using an in vitro digestion model under fed conditions. The digestion model consists of initial saliva processing for 5 min at 37 °C to simulate the mouth compartment and the gastric conditions for 2 h, followed by simulated small intestine compartment for 2 h at 37 °C [1]. AFs and OTA-free samples were spiked with AFB_1_ and OTA at two levels (10 and 5 μg/g) in single and combination. The extraction procedure was based on a mixture of acetonitrile/water (84/16, v/v) as described by Juan et al. [2]. Analytes were determined by liquid chromatography coupled to triple quadrupole mass spectrometry (LC-MS/MS). The bioaccessibility from bread matrices ranged from 68 to 45% for OTA, and 40 to 34% for AFB_1_, respectively. AFB_1_´s results were similar and slightly higher than observed in previous studies at 54 and 26% [3]. It highlights that there are few studies on AFB_1_ and OTA bioaccessibility, which are valuable to correlate with the real exposure risks and permits to conclude bioavailability studies. Therefore, the present study is included in a current in-progress bioavailability study.

**Keywords:** bread; mycotoxins; digestion; OTA; AFB_1_

References

1. Minekus M.; Alminger, M.; Alvito, P.; Ballance, S.; Bohn, T.; Bourlieu, C.; Carrière, F.; Boutrou, R.; Corredig, M.; Dupont, D.; et al. A standardised static in vitro digestion method suitable for food—an international consensus. *Food Funct.*
**2014**, *5*, 1113–1124.
2. Juan, C.; Ritienib, A.; Mañesc, J. Occurrence of *Fusarium* mycotoxins in Italian cereal and cereal products from organic farming. *Food Chem.*
**2013**, *141*, 1747–1755.
3. Saladino, F.; Posarelli, E.; Luz, C.; Luciano, F.B.; Rodriguez-Estrada, M.T.; Mañes, J.; Meca, G. Influence of probiotic microorganisms on aflatoxins B1 and B2 bioaccessibility evaluated with a simulated gastrointestinal digestion. *J. Food Compos. Anal.*
**2018**, *68*, 128–132.

### 4.11. Novel Bio(Mimetic) Recognition Elements for Optical Sensing and Analytical Separations of Mycotoxins in Foodstuff

Benito-PeñaElena^1^UrracaJavier^1^DescalzoAna Belén^2^PeltomaaRiikka^1^Luque-UríaAlvaro^1^QuilezJosé^2^Gómez-ArribasLidia N.^1^Glahn-MartínezAna B.^1^PradanasFernando^1^CalahorraLuis^2^MonterdeCristina^2^SerranoLuis Antonio^2^OrellanaGuillermo^2^*Moreno-BondiMaría Cruz^1^*1Department of Analytical Chemistry, Faculty of Chemistry, Complutense University of Madrid, 28040 Madrid, Spain2Department of Organic Chemistry, Faculty of Chemistry, Complutense University of Madrid, 28040 Madrid, Spain*Correspondence: gorellana@quim.ucm.es (G.O.); mcmbondi@ucm.es (M.C.M.-B.)

New sensing strategies are demanded by European stakeholders to reduce the cost of analysis, to fulfill the increasingly stringent European regulations, and to satisfy the pressing consumer demand for safer food products. Our current research lines focus on the synthesis of novel selective (bio)mimetic recognition elements for developing optical biosensors and separation methods to satisfy mycotoxin analysis’s legal, economic, and societal needs. We have applied the phage display technique to the selection of mycotoxin peptide mimetics, or mimopeptides, that have led to optical biosensors and bioassays. Random 12-mer and cyclic 7-mer peptide libraries have allowed the identification of mycotoxin epitope mimics that might replace the conventional hapten conjugates in competitive immunoassays. We have identified mimopeptides of fumonisin B_1_, zearalenone, and HT-2 toxin that turned out to be excellent mimics for biosensing with microarrays, magnetic bead-based, and homogeneous immunoassays. The peptides have been fused by genetic engineering with fluorescent or bioluminescent proteins providing more simple alternatives for target analysis, as neither secondary antibodies nor further labeling are required for the assay. The novel platforms feature superb sensitivities (detection limits below the LMRs for all the mycotoxins tested) and have been validated using certified References materials. Molecularly imprinted polymers (MIPs) are synthetic antibody mimics that selectively recognize molecular targets. MIPs are highly robust materials, showing superior operational stability under a wide variety of conditions. We have developed selective MIPs for the analysis of zearalenone, alternariol, alternariol monomethyl ether, citrinin, and tenuazonic acid mycotoxins, exploring new sensing mechanisms for developing optical sensors and as selective solid-phase extraction sorbents for those mycotoxin analyses in foodstuff. All these approaches have demonstrated the applicability of MIPs in the field of food safety and quality assurance and control.

**Keywords:** bio(mimetic); molecularly imprinted polymers; phage display; optical sensors; solid-phase extraction

**Acknowledgments:** Spanish Ministry of Science, Innovation and Universities (grant no. RTI2018-096410-B-C21/22). R. P. thanks UCM for a postdoctoral research contract, A.L.U. and J. Q thank the Ministry of Economy, Industry and Competitivity for a FPI grant.

### 4.12. Improved Method for the Determination of Ergot Alkaloids in Cereals and Cereal-Based Products

Carbonell-RozasLaura^1^*MahdjoubiChoukri Khelifa^2^Gámiz-GraciaLaura^1^Arroyo-ManzanaresNatalia^3^LaraFrancisco J.^1^García-CampañaAna M.^1^1Department of Analytical Chemistry, Faculty of Sciences, University of Granada, 18071 Granada, Spain2Department Biology, Faculty of Natural and Life Science, University of Oran 1, 31100 Oran, Algeria3Department Analytical Chemistry, Faculty of Chemistry, University of Murcia, 30003 Murcia, Spain*Correspondence: rozas@ugr.es

Ergot alkaloids (EAs) are toxins produced mainly by fungi of the *Claviceps* genus, prevalent in cereals, whose ingestion can cause ergotism. Although the European Commission has established a maximum content of 0.5 g/kg of ergot sclerotia in most unprocessed cereals, the maximum content for EAs in food is still under study. QuEChERS methodology has been widely applied for the multi-class analysis of mycotoxins in cereals, but EAs have been rarely included. In this work, an improved QuEChERS-UHPLC-MS/MS method is proposed to determine the major EAs [ergometrine (Em), ergosine (Es), ergotamine (Et), ergocornine (Eco), ergokryptine (Ekr), ergocristine (Ecr)], and their corresponding epimers [ergometrinine (Emn), ergosinine (Esn), ergotaminine (Etn), ergocorninine (Econ), ergokryptinine (Ekrn) and ergocristinine (Ecrn)] in cereals and cereal-based products. To improve sensitivity and to reduce organic solvent consumption, only 4 mL of acetonitrile and 3.0 mmol/L ammonium carbonate (85:15, *v*/*v*) were used as extractant. Moreover, 150 mg of Z-Sep+/C18 were selected as dispersive sorbents, decreasing matrix effect (<20% for most EAs) and improving recoveries (between 85–109%). Extraction under alkaline conditions as well as the rapid sample treatment enabled to minimize the EAs epimerization during the procedure. Procedural calibration curves were established for each matrix; LOQs were below 3.9 μg/kg in all cases, significantly lower in comparison with those obtained from other procedures. Precision (intra and inter-day) was lower than 15% (RSD) in all cases. Finally, 85 cereal samples were analyzed (30 of barley, 30 of wheat, and 25 of oat-based products), with the following positive results: One oat bran sample (total EAs content of 10.7 μg/kg), four barley samples (total EAs content between 18.0–54.0 μg/kg), and eight wheat samples (total EAs contents between 6.5–77.4 μg/kg). Em was the most frequently found, being in 70% of the positive samples.

**Keywords:** ergot alkaloids; cereals; QuEChERS; liquid chromatography; tandem mass spectrometry

**Acknowledgments:** Spanish Ministry of Science, Innovation and Universities (Project Ref.: RTI2018-097043-B-I00).

### 4.13. Potential of Metagenomic Approaches to Study Mycotoxigenic Fungal Diversity and Biocontrol Agent Cantidates in Soil Vineyars

Gil-SernaJéssica*Sáez-MatíaAlbaVázquezCovadongaPatiñoBelénDepartment of Genetics, Physiology and Microbiology, Faculty of Biology, Complutense University of Madrid, 28040 Madrid, Spain*Correspondence: jgilsern@ucm.es

Soil is the main reservoir of mycotoxigenic fungi in vineyards, and the application of biocontrol agents (BCA) might be a good option to reduce mycotoxin in grapes and their derivatives. This fact is especially relevant in organic management because the use of conventional fungicides is not permitted.

In this work, we performed a metagenomic approach to unveil the differences between organic and conventionally managed vineyards regarding (i) the diversity of soil microbiota, (ii) the occurrence of mycotoxigenic species, and (iii) the presence of potential BCA candidates. Ten samples from vineyard soils were collected from 5 locations in Spain. Next-generation sequencing was performed using 16S and ITS2 libraries.

The analysis of Shannon and Inverse Simpson indexes were used to evaluate diversity and species richness. In all cases, their values were related to rich communities, which are uniform, and no species were found to be dominant.

Potential mycotoxigenic species were present in all samples, although no evident relationships were found between management or location and contamination levels. At least two potential mycotoxigenic *Aspergillus* species were detected in all samples, being *Aspergillus niger* aggregate and *Aspergillus flavus* the most frequently occurring. However, the most unexpected result was the presence of high levels of *Fusarium incarnatum-equiseti* species complex since its relevance has not been previously reported in grapes and derivatives.

Potential fungal and bacterial BCA were found in all soil samples regardless of vineyard management. It is important to highlight the high occurrence of the actinomycetes *Pseudoarthrobacter* in all samples as well as *Hanseniaspora uvarum* that represented more than 50% of the fungal diversity in a sample collected in a conventional vineyard from Valencia.

These results revealed the potential of metagenomics to perform integrated approaches to study soil communities and their relation to mycotoxigenic species and potential BCA.

**Keywords:** next-generation sequencing; biological control; soil microbiota; diversity index

**Acknowledgments:** The authors thank the AgroBank-UdL Chair “Quality and Innovation in the Agri-Food Sector” for economic aid for the realization of this work.

### 4.14. Antifungal Activity of Peracetic Acid against Aspergillus flavus in Corn

QuilesJuan Manuel*CarbonellRaquelMañesJordiMecaGiuseppeLaboratory of Food Chemistry and Toxicology, Faculty of Pharmacy, University of Valencia, 46100 Burjassot, Valencia, Spain*Correspondence: Juan.quiles@uv.es

Peracetic acid (APA) is a compound used in the food industry for the disinfection of food and food-contact surfaces. Its mechanism of action is to oxidize lipid membranes, DNA, metabolites, and proteins with sulfhydryl groups and double bonds. Its main advantages are its wide antimicrobial spectrum and that its decomposition products (CH_3_COOH, O_2_, and H_2_O) are substances with low toxicity.

This study aims to quantify the antifungal activity of APA against the toxigenic fungi *Aspergillus flavus* (*A. flavus*), a common corn contaminant. First, the Minimum Inhibitory Concentration (MIC) and Minimum Fungicidal Concentration (MFC) of APA were established against *A. flavus* in liquid medium, using the 96-well plate method. Secondly, the use of APA released through a hydroxyethyl cellulose (HEC) gel in 1 L jars was tested to inhibit the growth of different concentrations of *A. flavus* inoculated into PDA solid media plates. Micellar growth was observed for 10 days. Finally, the same methodology was used to evaluate the antifungal activity and the reduction of aflatoxin B1 (AFB1) production caused by the APA-releasing HEC gel in *A. flavus* contaminated corn. Fungal growth was studied by colony counting in PDA plates seeded with decimal serial dilutions of contaminated corn; AFB1 production was determined in methanol extracted samples of contaminated corn analyzed by mass spectrometry-associated liquid chromatography (HPLC-MS/MS).

The results of MIC and MFC for APA against *A. flavus* in liquid medium were 125 mg/L and 187.5 mg/L, respectively. The antifungal doses for APA released through an HEC gel were estimated in the range between 10 mg/L and 25 mg/L for *A. flavus* inoculated in PDA plates and 300 mg/L for the contaminated corn, where this dose was also able to reduce AFB1 production completely.

**Keywords:** aflatoxin B1; antifungal activity; *Aspergillus flavus*; minimal inhibitory concentration; minimal fungicide concentration

### 4.15. The Occurrence of Aflatoxins in Nuts and Dried Fruits from Algeria

BelasliAzem^1^DjenaneDjamel^1^HerreraMarta^2^AriñoAgustín^2^*1Laboratoire de Qualité et Sécurité des Aliments, Département Technologie Alimentaire, Université Mouloud MAMMERI de Tizi-Ouzou, 15000 Tizi-Ouzou, Algeria2Veterinary Faculty, Instituto Agroalimentario de Aragón–IA2, University of Zaragoza–CITA, 50013 Zaragoza, Spain*Correspondence: aarino@unizar.es

The aflatoxins AFB1, AFB2, AFG1, and AFG2 are mycotoxins produced primarily by toxigenic strains of the fungi *Aspergillus flavus* and *Aspergillus parasiticus*. Aflatoxin-producing fungi are found in areas with a hot, humid climate, and aflatoxins in food are a result of both pre- and post-harvest fungal contamination. The aflatoxins are genotoxic, and they are classified by IARC as Group 1 (carcinogenic to humans). Maximum levels are set for AFB1 and the sum of AFB1, AFB2, AFG1, and AFG2 in tree nuts, groundnuts (peanuts), and other oilseeds, dried fruits, cereals, and some spices as well as processed products thereof. For AFB1, maximum levels are also set for baby foods and processed cereal based foods for infants and young children, as well as in dietary foods for special medical purposes intended especially for infants.

This study was conducted to screen the occurrence of the 4 aflatoxins in 114 samples (24 peanuts, 21 almonds, 20 walnuts, 29 dried figs, and 20 dates) purchased in retail shops and local markets in different regions from Algeria during 2019. The contamination levels were assessed, after an immunoaffinity column cleanup, by high-performance liquid chromatography coupled to fluorescence detector using photochemical derivatization (HPLC-PHRED-FLD). The LOD and the LOQ of the used method were 0.02 and 0.06 μg/kg, respectively. The results revealed that 27 out of 114 samples (23.7%) were positive for at least one aflatoxin. About 22 samples (19.3%) were contaminated by AFB1, with levels ranging from 0.02 to 5.25 μg/kg. The most frequently found aflatoxin in contaminated food samples was AFB1, and the three others were generally not reported in the absence of AFB1.

**Keywords:** aflatoxins; nut; dried fruits; HPLC

**Acknowledgments:** The authors thank the financial support of the Ministry of Higher Education and Scientific Research of Algeria (Grant D00L01UN150120180002) and the Government of Aragón and FEDER 2014-2020 (Grant Grupo A06_20R).

### 4.16. Transcriptome Analysis of Non-Ochratoxigenic Aspergillus carbonarius Strains

CastelláGemma^1^*BragulatM. Rosa^1^CiglianoRiccardo Aiese^2^CabañesF. Javier^1^1Veterinary Mycology Group, Department of Animal Health and Anatomy, Veterinary Faculty, Autonomous University of Barcelona, 08193 Barcelona, Spain2Sequentia Biotech S.L., 08005 Barcelona, Spain*Correspondence: Gemma.Castella@uab.cat

*Aspergillus carbonarius* consistently produces large amounts of ochratoxin A (OTA), a mycotoxin with nephrotoxic effects on animals and humans. In previous studies, we resequenced the genome of three atypical and unique non-OTA-producing strains of *A. carbonarius* [1,2]. Although no large deletions in functional genes related to OTA production were found, some private missense variants of non-ochratoxigenic strains in the *AcOTApks* gene were detected [2]. The rest of the OTA cluster genes (*AcOTAnrps*, *AcOTAp450*, *AcOTAhal*, and *AcOTAbZIP*) showed no privative mutations in the non-OTA-producing strains. In this study, we applied the RNA Sequencing technology to carry out a global transcriptional analysis on four *A. carbonarius* strains, one OTA producer, and the three atypical non-OTA-producing strains, and to analyze the differentially expressed genes directly or indirectly related to OTA biosynthetic pathway [3]. A total of 696 differentially expressed genes (DEGs) were identified comparing the OTA-producing strain vs. the three non-OTA-producing strains. Among these DEGs, 280 genes were up-regulated, and 333 genes were down-regulated in the non-OTA-producing strains. Among down-regulated DEGs, over-represented biological process categories were oxidation-reduction and metabolic processes. Where molecular function is concerned, oxidoreductase and hydrolase activities were over-represented. Within the most down-regulated genes in the three non-OTA-producing strains, we found the *AcOTApks* and *AcOTAnrps* genes. The *AcOTAp450* gene the transcription factor *AcOTAbZIP* were also down-regulated. Besides, the *AcOTAhal* gene was down-regulated, although no significant differences were observed. We also confirmed the five common missense variants found in the *AcOTApks* gene of these atoxigenic strains, and we showed that one of these mutations can have a deleterious impact on the biological function of the acyltransferase domain of this gene.

**Keywords:***Aspergillus carbonarius*; ochratoxin A; transcriptome

**Acknowledgments:** This research was supported by the Ministerio de Economía y Competitividad of the Spanish Government (AGL2014-52516-R).

References

1. Cabañes, F.J.; Sanseverino, W.; Castellá; G; Bragulat, M.R.; Cigliano, R.A.; Sánchez, A. Rapid genome resequencing of an atoxigenic strain of *Aspergillus carbonarius*. *Sci. Rep.*
**2015**, *5*, 9086.
2. Castellá, G.; Bragulat, M.R.; Puig, L.; Sanseverino, W.; Cabañes, F.J. Genomic diversity in ochratoxigenic and non ochratoxigenic strains of *Aspergillus carbonarius*. *Sci. Rep.*
**2018**, *8*, 5439.
3. Castellá, G.; Bragulat, M.R.; Cigliano, R.A.; Cabañes, F.J. Transcriptome analysis of non-ochratoxigenic *Aspergillus carbonarius* strains and interactions between some black aspergilli species. *Int. J. Food Microbiol.*
**2020**, *317*, 108498.

### 4.17. Regulation of Secondary Metabolism in Aspergillus carbonarius

LlobregatBelénGonzález-CandelasLuisBallesterAna-Rosa*Institute of Agrochemistry and Food Technology (IATA-CSIC), 46980 Paterna, Spain*Correspondence: ballesterar@iata.csic.es

*Aspergillus carbonarius* is one of the main species responsible for toxin contamination of grapes and their derivatives, wine, coffee, and cocoa. Ochratoxin A (OTA), the major mycotoxin produced by *A. carbonarius*, is a secondary metabolite classified as a possible human carcinogen due to its high nephrotoxic character and the immunosuppressive effects that it triggers. It is known that growth, development, and secondary metabolite production are interconnected processes controlled by global regulatory factors in filamentous fungi. Besides that, these regulatory factors are encoded by genes that are generally located outside the gene clusters involved in the biosynthesis of secondary metabolites. An example of these global regulatory factors is the VELVET Complex (VelB/VeA/LaeA), a complex that couples fungal development with secondary metabolism, in response to light. Studies have demonstrated the relation between the loss of *veA* and *laeA* and the drastic reduction of OTA and conidial production. One of the biosynthetic genes within the putative OTA cluster of *A. carbonarius* is the polyketide synthase *pks* gene. In the present study, we have independently deleted *veA*, *laeA* and *pks* genes in the ochratoxigenic *A. carbonarius* ITEM 5010 strain by targeted gene replacement using *Agrobacterium tumefaciens*-mediated transformation. Finally, we are developing the CRISPR-Cas9 system mediated by *A. tumefaciens* transformation by using the *alb1* gene as a phenotypic marker because the deletion of this gene produces white spores.

**Keywords:** CRISPR-Cas9; gene editing; *Agrobacterium tumefaciens* mediated transformation; ochratoxin A

**Acknowledgments:** This work was supported by the Spanish Ministry of Science, Innovation and Universities (RTI2018-093392-A-I00, AEI/FEDER, UE). B. Llobregat is the recipient of a JAEIntro2019-CSIC (JAEINT_19_01896) and a predoctoral contract (PRE2019-089326) and A.-R. Ballester is the recipient of a Ramon y Cajal contract (RYC-2017-22009).

### 4.18. In Vitro Biocontrol of Aspergillus carbonarius and Ochratoxin A by Bacillus spp.

EinloftTiago C.*de OliveiraPatricia BolzanDionelloRafael GomesDepartamento de Fitossanidade, Universidade Federal do Rio Grande do Sul, CEP: 90040-060 Porto Alegre, RS, Brazil*Correspondence: tiago.einloft@gmail.com

*Aspergillus carbonarius* is the main Ochratoxin A (OTA) producer in grapes destined for wine production. Recently, biological control methods have been used due to their high efficiency, the absence of resistance development, and low environmental impact. In this study, we screened the biocontrol capabilities of three Bacillus isolates, which previously showed antagonistic proprieties towards other mycotoxigenic fungi, towards *A. carbonarius* growth and OTA production. Bacterial suspensions were mixed with molten Czapek yeast extract (CYA) medium and pour plated into petri dishes. After solidification, an agar plug of *A. carbonarius* was inserted in the center of the plate. Cultures were incubated at 25 °C for 7 days and the radial mycelium was measured daily to determine mycelial growth rate. Subsequently, the colonies were washed twice with aqueous tween 80, and the number of conidia was determined. To evaluate the biocontrol effect on OTA production, both microorganisms were inoculated in CYA broth, which was incubated at 25 °C for 7 days on a rotary shaker. After incubation, 10 mL aliquots of the culture were removed, and OTA was extracted with chloroform. The extracts were analyzed by UPLC/MS-MS. *A. carbonarius* mycelial growth rate was drastically reduced by all bacteria, ranging from 76% to 96% of inhibition. Conidial production was also significantly reduced by all three bacteria. Bacillus sp. RP103 and RP242 were most effective, reducing conidial production by 10 times. OTA production by *A. carbonarius* was reduced in a range from 86 to 93%. The results obtained in this study indicate that these bacteria have promising biocontrol characteristics and could characterize a new strategy for the management of *A. carbonarius* growth and OTA contamination in wine grapes.

**Keywords:** biocontrol; *Aspergillus carbonarius*; *Bacillus*; ochratoxin A; wine grapes

### 4.19. Evaluation of Plant Extracts and Protective Cultures Effects on Ochratoxigenic Penicillium nordicum in a Dry-Cured Fermented Sausage Model

RonceroElia*DelgadoJosuéRomeroPaulaPulidoJuan CarlosAndradeMaría JesúsFood Hygiene and Safety, Meat and Meat Products Research Institute, Faculty of Veterinary Science, University of Extremadura, 10003 Cáceres, Spain*Correspondence: eroncerob@unex.es

The surface of dry-cured fermented sausages is colonized during their ripening by molds, being some of them ochratoxigenic. Nowadays, the meat industry uses synthetic antifungal compounds to avoid the growth of such molds, together with other unwanted ones. Nevertheless, several strategies based on using natural preservatives, such as plant extracts and native microorganisms, are gaining interest. The aim of this study was to evaluate the antagonist activity of the essential oil of rosemary (REO), a spice commonly used in the dry-cured fermented sausages processing, and the acorn extract (AE) in the presence and absence of a *Debaryomyces hansenii* strain with known ability to be used as a protective culture. Slices of raw dry-cured fermented sausages “chorizo” were thus placed in pre-sterilized receptacles with 86% relative humidity. After inoculating them with the treatments alone and in combination, three ochratoxigenic strains of *Penicillium nordicum* (PN856, PN92, and PN15) were inoculated. As a negative control, only the molds were separately inoculated. After incubating all the treatments at 12 °C for 15 days, ochratoxin A (OTA) was quantified using an uHPLC coupled to a Q exactive Plus detector. Different patterns in the ability of the biocontrol agents to alter the OTA production were detected at the mold strain level. In PN92 and PN15, a significant positive synergic effect was detected in the presence of AE and the yeast. Similarly, the combination of AE with REO significantly reduced the OTA level in PN15. Nonetheless, REO was the unique treatment provoking a significant reduction of OTA production in PN856. When REO, AE, and the yeast were inoculated together, a significant increase in the OTA levels was observed in PN92 and PN15. Consequently, further studies to evaluate increasing concentrations of the biopreservative agents without modifying the sensorial characteristics of the products and able to reduce the OTA production in all the ochratoxigenic strains should be developed.

**Keywords:** ochratoxin A; plant extracts; dry-cured fermented sausages; antagonist activity

**Acknowledgments:** This research was funded by Junta de Extremadura and Fondo Europeo de Desarrollo Regional—“Una manera de hacer Europa” (IB16045 project and GR18056 grant).

### 4.20. Biocontrol of Penicillium nordicum in Dry-Cured Fermented Sausages

ÁlvarezMicaela*GómezFranciscoAsensioMiguel ÁngelBermúdezElenaNúñezFélixUniversity of Extremadura, Institute of Meat and Meat Products, Faculty of Veterinary Science, Food Hygiene and Safety, 10003 Cáceres, Spain*Correspondence: maalvarezr@unex.es

*Penicillium nordicum* is the main ochratoxin A (OTA) producer in dry-cured fermented sausages. Since the consumers demand additive-free products, strategies to avoid OTA presence are nowadays focused on vegetal and microbial agents. Previous in vitro studies have shown efficiency for reducing OTA of rosemary leaves (RL), rosemary essential oil (REO), and *Debaryomyces hansenii* (Dh). The objective of the study was to test their antifungal effect when applied alone or in combination. Dry-cured fermented sausages were processed in a pilot plant using 18 combinations of these biocontrol agents. Batch 1 was uninoculated with them. Batch 2 included 2 g/kg of RL added to the meat mix before stuffing. In batch 3, the casings were macerated in water with RL for one day before stuffing. In batch 4, REO was applied after stuffing. Batch 5 included a combination of REO and RL. In batch 6, a commercial antifungal (natamycin + potassium sorbate) was applied. Besides, Dh was applied together with the other treatments inside the meat (batches 7–12) and after stuffing (batches 13–18). *P. nordicum* was finally inoculated on the surface of all treated sausages. After ripening, OTA was quantified by UHPLC-MS/MS. Significant reductions of OTA presence due to most of the treatments with RL and Dh in the meat mix (batches 2, 3, 7, 8, 9, 11f, and 12) were detected, being the majority of the obtained levels below the limit of quantification (<0.25 ng/g). The presence of the yeast on the surface only reduced OTA levels when it was inoculated alone (batch 13), with RL (batches 14 and 17) and with the commercial antifungal (batch 18). Therefore, the incorporation of rosemary and *D. hansenii* in the meat mix before stuffing can be successfully implemented as synergic antifungal agents during the processing of dry-cured fermented sausages.

**Keywords:** rosemary; essential oil; Debaryomyces hansenii; ochratoxin A; dry-cured fermented sausages

**Acknowledgments:** This research was funded by Junta de Extremadura-Consejería de Economía, Ciencia y Agenda Digital-, Fondo Europeo de Desarrollo Regional-“Una manera de hacer Europa” (IB16045 project and GR18056 grant). M. Álvarez is recipient of a fellowship from the Spanish Ministerio de Economía, Industria y Competitividad (BES-2017-081340).

### 4.21. Switching Energy Metabolism Increases Susceptibility of SH-SY5Y Cells to Sterigmatocystin: A Mitochondrial Toxin

ZingalesVeronica*Fernández-FranzónMónicaRuizMaría JoséLaboratory of Toxicology, Faculty of Pharmacy, University of Valencia, 46100 Burjassot, Valencia, Spain*Correspondence: vezin@uv.es

Mitochondria are key cellular organelles known to guarantee many physiological processes, such as energy production through the oxidative phosphorylation process (OXPHOS). In in vitro conditions, cell lines are metabolically adapted to grow rapidly, and, for this reason, they derive most of their energy from glycolysis rather than OXPHOS, a phenomenon known as the Crabtree effect. The substitution of galactose for glucose in the culture medium is an expeditious way to reverse the Crabtree effect and determine mitochondrial toxicity. The aim of the present study was to evaluate the role of mitochondria in the toxicity induced by the mycotoxin sterigmatocystin (STE) on human neuroblastoma SH-SY5Y cells. Cells were cultured in the presence of glucose (25 mM) or galactose (10 mM) as the only sugar available. The effects on cell viability were evaluated by MTT assay. The change in the fuel source caused a cell viability decrease on galactose-grown cells compared to glucose-grown cells, suggesting that STE exhibited an increased level of toxicity in SH-SY5Y cells following the switch to OXPHOS. Furthermore, considering the crucial importance of a functional electron transport chain (ETC) in OXPHOS conditions, we also compared the effect of STE exposure in the presence or not of known ETC inhibitors (antimycin A and rotenone) in cells grown in a galactose-supplemented medium. Treatment with STE and rotenone, a selective inhibitor of the complex I, resulted in a further significant decrease in cell viability with respect to cells only exposed to the mycotoxin, while no effect was observed in the presence of antimycin A, an inhibitor of the complex III. These data highlight that STE might affect complex I, whereas the complex III of the ETC seems not to be involved in STE toxicity. Taken together, our results suggest that the etiology of STE cytotoxicity may depend on mitochondrial impairment.

**Keywords:** sterigmatocystin; SH-SY5Y cells; cytotoxicity; mitochondria; Crabtree effect

**Acknowledgments:** This research has been supported by the Generalitat Valenciana grant (Prometeo 2018/216) and the pre-doctoral research training program “Santiago Grisolia (GRISOLIAP/2018/092) CPI-18-117”.

### 4.22. Biomonitoring of Mycotoxins in Plasma of Spanish Adults

Arce-LópezBeatriz*IrigoyenÁngelLizarragaElenaGonzález-PeñasElenaPharmaceutical Technology and Chemistry Department, Research Group MITOX, Faculty of Pharmacy and Nutrition, University of Navarra, 31008 Pamplona, Spain*Correspondence: barce@alumni.unav.es

Human biomonitoring has been considered an efficient way to assess human exposure to mycotoxins. Adequate and validated analytical methods are crucial for this purpose. Furthermore, it is necessary to select good biomarkers in biological fluids [1], and plasma seems to be a suitable matrix to perform these studies.

The aim of this study is to assess mycotoxin exposure through the analysis of 19 compounds in plasma samples from a region of northern Spain. The impact of some factors (age and gender) on this exposure has also been evaluated. Plasma from healthy adults (*n* = 438, aged 19–68 years) was analyzed using an LC-MS/MS validated method [2]. Sample preparation was carried out by deproteinization and cleanup using Captiva EMR^®^-lipid (3 mL) cartridges. In order to study the presence of some Phase II metabolites, plasma samples were treated with a mixture of β-glucuronidase/arylsulfatase enzymes.

The most prevalent mycotoxin was ochratoxin A (OTA), with an incidence of 97.3% and positive samples are in the range of 0.4 to 45.7 ng/mL (0.4–23.3 ng/mL after enzymatic treatment) with a mean value of 2.87 ng/mL (2.40 ng/mL after enzymatic treatment). Ochratoxin B has also been detected (10% of the samples), and its presence can be related to that of OTA. Sterigmatocystin was detected in 85.8% of the samples, only after enzymatic hydrolysis, supporting conjugation as a pathway of its metabolism in humans. None of the other studied mycotoxins (aflatoxins B1, B2, G1, G2, and M1; T-2 and HT-2 toxins; deoxynivalenol, deepoxy-deoxynivalenol, 3-acetyldeoxynivalenol, 15-acetyldeoxynivalenol; zearalenone; nivalenol; fusarenon-X; neosolaniol; and diacetoxyscirpenol) were detected in any of the samples, neither before nor after enzymatic treatment. To the best of our knowledge, this is the first study carried out in Spain to determine multi-mycotoxin exposure through their presence in human plasma [3].

The present biomonitoring study generates reliable and critical data regarding the exposure of the Spanish population to mycotoxins.

**Keywords:** human plasma biomonitoring; mycotoxins; ochratoxin A; ochratoxin B; sterigmatocystin

**Acknowledgments:** This work was supported by the Spanish “Ministerio de Economía, Industria y Competitividad, Agencia Estatal de Investigación” (AGL2017-85732-R, MINECO/AEI/FEDER, UE).

References

1. Arce-López, B.; Lizarraga, E.; Vettorazzi, A.; González-Peñas, E. Human Biomonitoring of Mycotoxins in Blood, Plasma and Serum in Recent Years: A Review. *Toxins*
**2020**, *12*, 147.
2. Arce-López, B.; Lizarraga, E.; Flores-Flores, M.; Irigoyen, Á.; González-Peñas, E. Development and validation of a methodology based on Captiva EMR-lipid clean-up and LC-MS/MS analysis for the simultaneous determination of mycotoxins in human plasma. *Talanta*
**2020**, *206*, 120193.
3. Arce-López, B.; Lizarraga, E.; Vettorazzi, A.; González-Peñas, E. Presence of 19 Mycotoxins in Human Plasma in a Region of Northern Spain. *Toxins*
**2020**, *12*, 750.

### 4.23. Does Mycotoxin Exposure Alter Brain Cells Differentiation in Vitro? Protocol Design and Optimization Using SH-SY5Y Cell Line

FrangiamoneMassimo*Alonso-GarridoManuelFontGuillerminaManyesLaraLaboratory of Food Chemistry and Toxicology, Faculty of Pharmacy, University of Valencia, 46100 Burjassot, Valencia, Spain*Correspondence: Massimo.frangiamone@ext.uv.es

Human SH-SY5Y neuroblastoma cells represent a suitable in vitro model to investigate toxicity in the brain and reproduce accurately neurodegenerative diseases. Although SH-SY5Y cells are widely used in neuronal research, they are epithelial cells with no neuronal proprieties unless they are treated with retinoic acid (RA) and differentiated into dopaminergic neurons. Thus, neuronal features, discriminating undifferentiated and RA-differentiated SH-SY5Y cells and showing significant differences between these cell models, will be characterized. In this regard, two different techniques, flow cytometry, and microscopy are being implemented to highlight morphological and functional changes induced by differentiation. Cells will be exposed to several mycotoxins concentrations, individually and in combination, and the exposure will start at different differentiation time points in order to assess the risk of exposure to these food contaminants. Flow cytometry experiment allows outlining differences in the cell cycle since differentiated cells featured a significant decrease in the proliferation rates, mainly associated with a decrease in S phase in combination with an arrest in G2-M. In this case, DNA composition will be analyzed using propidium iodide. Instead, optical microscopy enables analysis of the cell morphology since RA-differentiated cells showed an increased neurite density, suggesting a change from epithelial to a stellate neuronal morphology. Moreover, immunofluorescence microscopy permits detection of the dopamine content, potentiated by RA-differentiation. Neurites density and dopamine reactivity will be evaluated using anti-bIII tubulin and anti-dopamine antibodies, respectively, followed by incubation with their Alexa 488-conjugated antibodies and nuclear dye Hoechst, thus achieving random images with a fluorescence microscope. Hence, through three different experiments: Cell cycle and cellular growth, dopamine immunoreactivity, and neurite density differentiation, alterations induced by the presence of mycotoxins will be investigated.

**Keywords:** SH-SY5Y; flow cytometry; microscopy; retinoic acid; differentiation

**Acknowledgments:** Spanish Ministry of Science and Innovation Project (PID2019-108070RB-I00-ALI) and PhD grant (BES-2017-081328).

### 4.24. Study of Enzymatic Defense System in Neuroblastoma Cells Exposed to Zearalenone’s Metabolites and Beauvericin

AgahiFojan*Juan-GarcíaAnaJuanCristinaLaboratory of Toxicology, Faculty of Pharmacy, University of Valencia, 46100 Burjassot, Valencia, Spain*Correspondence: fojan@alumni.uv.es

Beauvericin (BEA), α-zearalenol (α-ZEL), and β-zearalenol (β-ZEL) are produced by several *Fusarium* species that contaminate cereal grains. These mycotoxins can cause cytotoxicity and genotoxicity in various cell lines, and they are also capable of produce oxidative stress at the molecular level. However, mammalian cells are equipped with a protective endogenous antioxidant system formed by non-enzymatic antioxidant and enzymatic protective systems such as glutathione peroxidase (GPx), glutathione S-transferase (GST), superoxide dismutase (SOD), and catalase (CAT).

The aim of this study was evaluating the effects of α-ZEL, β-ZEL, and BEA, on enzymatic GPx, GST, SOD, and CAT activity in human neuroblastoma cells using the SH-SY5Y cell line, over 24 h and 48 h with individual treatment at the concentration range from 1.56 to 12.5 μM for α-ZEL and β-ZEL, from 0.39 to 2.5 μM for BEA, from 1.87 to 25 μM for binary combinations and from 3.43 to 27.5 μM for tertiary combination.

Our results revealed a significant increase in GPx activity after 24 h of exposure in all treatments except for tertiary combination, which decreased notably; while after 48 h, only BEA and triple mixture increased GPx activity considerably. GST activity in SH-SY5Y cells decreased significantly after exposing them to α-ZEL and β-ZEL, while in combinations increased notably after 24 h, except for β-ZEL + BEA, where a considerable decrease at lowest concentrations and increase at highest concentrations was detected. After 48 h, a significant increase in β-ZEL and BEA, whereas a decrease in α-ZEL + β-ZEL combination was observed. CAT activity decreased significantly in all treatments after 24 h except in β-ZEL + BEA, which revealed an increase. For SOD, no changes were observed after 24 h, although a significant increase was observed in binary combinations α-ZEL + BEA and β-ZEL + BEA after 48h, respectively.

**Keywords:** enzymatic defense; SH-SY5Y cells; zearalenone derivates; beauvericin

### 4.25. Proteomic Changes Associated with Enniatins Acute Exposure in Rat Liver

CimbaloAlessandra*LozanoManuelJuanCristinaFontGuillerminaMan-yesLaraLaboratory of Food Chemistry and Toxicology, Faculty of Pharmacy, University of Valencia, 46100 Burjassot, Valencia, Spain*Correspondence: alscim@uv.es

Enniatins (ENs) are hexadepsipeptides produced by *Fusarium* fungi, which can act as ionophores, disturbing membrane homeostasis. In this study, a proteomic analysis to determine the acute response of rat’s liver to ENs exposure at different concentrations was carried out. A total of 14 female 2 months old Wistar rats were employed, divided into 3 groups. Five of the treated ones were intoxicated with medium concentrations: Single dose of ENA 256, ENA1 353, ENB 540, ENB1 296 µg/mL; and other five with the higher ones: Single dose of ENA 513, ENA1 706, ENB 1021, ENB1 593 µg/mL for 8 hours exposure. Protein extraction was performed using 10 mg of powdered liver tissue in an 8M Urea/2M Thiourea/50mM Tris-HCl lysis buffer. Protein concentration was determined by using a spectrophotometer NanoDrop™ 2000 and subsequently standardized to 1 mg/mL. Samples were mixed with dithiotreitol and iodoacetamide for alkylation of cysteine residues and digested with the addition of trypsin (1:40) overnight. Peptides were dried on a vacuum concentrator and eluted in 0.1 % acetic acid: Acetonitrile (98:2 *v*/*v*) to a final concentration of 100 μg/μL. Samples were analyzed using an LC system coupled with quadrupole time of flight (Q-TOF), and the obtained chromatograms were aligned with Mass Hunter Professional software (Agilent). Peptides identification was carried out by Spectrum Mill software and statistically filtered by abundance using Mass Professional Profiler software (Agilent). Results reported a total of 57 differentially expressed proteins in both medium and high treated animals when compared to the control. DAVID gene ontology analysis revealed acetylation, nucleotide phosphate-binding region:NAD, and catalytic activity as the most represented terms in the bioinformatics analysis. Moreover, 13 of these proteins were found in the mitochondrion, and 12 were related to oxidoreductase activity. Regarding reactome overrepresentation test results, metabolism was both the most significant pathway and the most enriched.

**Keywords:** mycotoxin; proteomics; Q-TOF; mitochondrion; metabolism

**Acknowledgments:** This work was supported by Spanish Ministry of Economy and Competitiveness (PID2019-108070RB-I00-ALI) and Generalitat Valencianan PhD grant (GVPROMETEO2018-126).

## 5. Poster Presentations

### 5.1. In Vitro Method Development for the Assessment of Ochratoxin and Aflatoxins Neurotoxicity and Mitigation Strategies with Carotenoids

Alonso-GarridoManuel*FrangiamoneMassimoFontGuillerminaManyesLaraLaboratory of Food Chemistry and Toxicology, Faculty of Pharmacy, University of Valencia, 46100 Burjassot, Valencia, Spain*Correspondence: Manuel.alonso-garrido@uv.es

Aflatoxins and ochratoxin neurotoxicity is a field of interest for food science researchers, as they are able to cross the blood–brain barrier, triggering mechanisms such as altered gene expression and oxidative stress, which are related to neurodegenerative disorders. On the contrary, carotenoids are known for their antioxidant capacity, and they have also been found in the brain. Thus, a method using SH-SY5Y neuroblastoma cells and low concentrations of mycotoxins (100 nM) and carotenoids (500 nM) is being developed simulating a real scenario in the human body. First, the effect of these mycotoxins on SH-SY5Y differentiation, individually and combined, will be assessed through optical, immunofluorescence, and confocal microscopy as well as flow cytometry. Optical microscopy will be used to visually discriminate non-differentiated cells, due to diverse morphology. Immunofluorescence will allow estimating neurite density through β-III tubulin detection, a marker for neuronal differentiation. Confocal microscopy will serve to detect dopamine immunoreactivity, which is also characteristic of neuronal cell activity. Second, Next Generation Sequencing (NGS) will be performed to find the most altered genes by the different cell exposures on an Illumina sequencer by RNA-seq technique. Data analysis will be performed on different bioinformatics tools for Differential Gene Expression (DEGs) and altered pathways. Third, validation of NGS will be done on the most affected genes by qPCR to confirm DEGs. Fourth, proteomics will be carried out in order to analyze if the profile is also modified, and these results will be compared with DEGs. Mass spectrometry technique (LC/Q-TOF MS) will allow to quantify the proteome followed by bioinformatics for peptide, protein identification and pathways involved.

**Keywords:** SH-SY5Y; ochratoxin a; aflatoxin; neurodegenerative disorders; omics

**Acknowledgments:** Spanish Ministry of Science and Innovation Project (PID2019-108070RB-I00-ALI) and grant (BES-2017-081328).

### 5.2. Biomonitoring Study of Citrinin and its Metabolite Dihydrocitrinone in Human Urine

NarváezAlfonso^1^*Rodríguez-CarrascoYelko^2^AlonsoCarmen Martínez^2^IzzoLuana^1^RitieniAlberto^1^1Department of Pharmacy, Università di Napoli Federico II, 80131 Napoli, Italy2Department of Food Chemistry and Toxicology, University of Valencia, 46100 Burjassot, Valencia, Spain*Correspondence: alfonso.narvaezsimon@unina.it

Citrinin is a mycotoxin produced by *Penicillium* and *Aspergillus* spp. with nephrotoxicity and genotoxicity attributed in human cell lines. Therefore, the European Food Safety Authority set a threshold of toxicological concern for citrinin at 0.2 μg/kg b.w., but the lack of data on food contamination hampers a proper exposure assessment. To overcome this, strategies based on the biomonitoring of citrinin in biological samples have become an alternative in order to assess human exposure. Since the toxins occur at very low concentrations in human samples, selective and sensitive methodologies are required for accurate measurements. Hence, the aim of this study was to evaluate the presence of citrinin and its metabolite dihydrocitrinone for the first time in 300 human urine samples from South Italy through an ultrahigh-performance liquid chromatography high-resolution mass spectrometry (UHPLC-Q-Orbitrap HRMS) methodology. Citrinin was quantified in 47% (*n* = 300) of samples with concentrations ranging from below the limit of quantification (LOQ = 0.012 ng/mL) to 4.003 ng/mL (mean value = 0.286 ng/mL), and dihydrocitrinone was detected in 21% of samples at levels from below the LOQ (0.012 ng/mL) up to 2.481 ng/mL (mean value = 0.386 ng/mL). These results are in accordance with previous works where the metabolite reflects higher average levels than the parental compound. Heavier contamination was observed when compared to other European countries, with a 6-to-10-fold increase of mean values, which could be explained by a higher intake of cereals within the Italian population. Statistical analysis revealed differences according to the age of the volunteers, with citrinin being significantly more present in the population from 30 to 60 years old that may be due to different dietary habits. These data reflect a high exposure to citrinin within the Italian population, supporting further toxicological and food safety investigations for a better understanding of its impact.

**Keywords:** citrinin; exposure assessment; biomonitoring; urine; Orbitrap

**Acknowledgments:** This research was funded by the project grant fiven by the Generalitat Valenciana (Spain) GV/2020/020 (Conselleria d’Innovacio, Universitats, Ciencia I Societat Digital, Generalitat Valenciana).

### 5.3. How Climate Change is Affecting to the Occurrence of Mycotoxins in Europe?

Vila-DonatPilar*TolosaJosefaRuizMaría JoséBerradaHoudaLaboratory of Food Chemistry and Toxicology, Faculty of Pharmacy, University of Valencia, 46100 Burjassot, Valencia, Spain*Correspondence: Maria.p.vila@uv.es

The impact of climate change on the presence of mycotoxins in food and feed is a topic of great concern. Mycotoxins are ubiquitously present in feeding stuff, being to date, *Fusarium* mycotoxins (deoxynivalenol, DON; zearalenone, ZEN; and fumonisins, FBs) prevalent in areas of temperate climates such as Europe and *Aspergillus* mycotoxins contamination more frequently in hot climates. Every mold species has its own optimum conditions of temperature and activity water or the growth and formation of mycotoxins, thus environmental factors such as high temperatures, high moisture levels, and insect damage contribute to the presence of mycotoxins in feeds. The aim of this work was to review recent data reported on the mycotoxin’s occurrence patterns in Europe because of environmental changes.

In the data obtained from the available literature, the evidence shows *A. flavus* infection and aflatoxin (AF) contamination, previously uncommon in Europe, would become increasingly important. Over the last decade, several hot seasons have led to severe *A. flavus* infections in maize in several European countries (Italy, Romania, Serbia, and Spain). AF outbreaks have been reported in some regions of South Europe, such as infection of maize by AFB1 in Italy from 2003 as a result of a hot and dry growing season. It is even being detected AFM1 in cow’s milk samples, in which differences depending on the season of sampling have been observed.

On the other hand, the prevalence of *Fusarium graminearum* (species adapted to hot conditions) and the main producer of DON in cereal grain, has already increased in Central Europe and is likely to increase in North Europe due to the expected changes in weather conditions.

These facts point out that occurrence patterns of mycotoxins in Europe are changing as a consequence of rising average temperatures and, consequently, a potential increase in consumer health risk in Europe.

**Keywords:** mycotoxins; climate change; occurrence patterns; Europe

**Acknowledgments:** Authors are grateful to Regional Government to fund GV/2020/020 project.

### 5.4. Exposure Assessment of Catalonian Population to Fusarium spp. Mycotoxins through Cereal-Based Food

GallardoJose A.^1^Cano-SanchoGerman^2^RamosAntonio J.^1^MarínSonia^1^SanchisVicente^1^*1Applied Mycology Group, University of Lleida, Agrotecnio Center, 25198 Lleida, Spain2Laboratoire d’Etude des Résidus et Contaminants dans les Aliments (LABERCA), Ecole Nationale Vétérinaire, Agroalimentaire et de l’Alimentation (Oniris), 44307 Nantes Cedex 3, France*Correspondence: vicente.sanchis@udl.cat

Mycotoxins produced by *Fusarium* genus may exert harmful effects on human health and, therefore, represent a hazard to public health and a challenge to ensure food safety. For this reason, the exposure assessment to deoxynivalenol (DON) and enniatin B (ENNB) through cereal-based food (e.g., bread, cookies, and pasta, among others) has been conducted. Consumption data have been gathered from surveys conducted in Catalonia during 2019, contamination data has been obtained from the analysis of the samples also collected in Catalonia. The data have been combined using three types of statistical approaches: A deterministic estimation, non-parametric estimation, and parametric estimation. In the case of infant exposure through baby formula, a deterministic estimation has been conducted due to a large number of censored samples. The results of these estimations (using the limit of detection to substitute censored data) showed the exposure of babies would be an average of 75 ng/kg bw/day for DON and 31 ng/kg bw/day for ENNB for the estimation through composite, man would be exposed to 102 ng/kg bw/day for DON and 83 ng/kg bw/day for ENNB, woman would be exposed to 100 ng/kg bw/day for DON and 82 ng/kg bw/day for ENN, and elderly would be exposed to 93 ng/kg bw/day for DON and 76 ng/kg bw/day for ENNB. The current exposure estimates for DON appeared to be similar to the previous assessment conducted in 2009, with the exception of a decrease among babies and a slight increase among elders, in any case far from the tolerable daily intake of 1000 ng/kg bw/day.

**Keywords:** deoxynivalenol; enniatin B; exposure; *Fusarium*

### 5.5. Evaluation of Mycotoxins in Infant Breast Milk

HernándezMarta*JuanCristinaDepartment of Preventive Medicine and Public Health, Food Sciences, Toxicology and Forensic Medicine, Faculty of Pharmacy, University of Valencia, 46100 Burjassot, Valencia, Spain*Correspondence: herher5@alumni.uv.es

A recent review of the presence of mycotoxins in breast milk published in the last years is presented. It has been carried out to provide an overview of infant population exposure by countries and continents.

OTA and AFM1 were the most present. The global mean and range detected were greatly different between continents and countries. A high incidence was observed in Tanzania, Iran, Jordan, and Turkey. However, the highest values were observed in Egypt, Sudan, and Serbia, being higher than the EU maximum limits (25 ng/kg for AFM1 and 500 ng/kg for OTA) (Regulation (EC) No 1881/2006). A provisional estimated daily intake (PDI) was calculated using the mean observed and an approximation of daily consumption of breast milk from 630 g/day to 890 g/day. EFSA (2006) established a tolerable daily intake (TDI) only for OTA with 14 ng/kg/day and non-intake for AFM1. It should be noted that the AFM1 values ranged from <1 ng/L to 7100 ng/L (Brazil and Egypt, respectively), then PDI was from 0.5 and 595 ng/kg bw/day. On the other hand, the range of OTA´s means were 4 to 1990 ng/L (Brazil and Iran) with an PDI from 0.38 to117 ng/kg bw/day, values that represent the 3 to 1194% of the TDI. The review indicates that more controls on raw materials should be applied in Egypt, Iran, and Turkey.

**Keywords:** mycotoxin; breast milk; infant; aflatoxin; ochratoxin A

### 5.6. Inhibition of Fusarium verticillioides in Tomato Fruits by Extracts of Trichoderma spp.

StracquadanioClaudia^1^,^2^*QuilesJuan Manuel^3^LuzCarlos^3^MecaGiuseppe^3^CacciolaSanta Olga^2^1Department of Agricultural Science, Mediterranean University of Reggio Calabria, 89123 Reggio Calabria, Italy2Department of Agriculture Food and Environment, University of Catania, 95131 Catania, Italy3Department of Preventive Medicine, University of Valencia, 46100 Burjassot, Valencia, Spain*Correspondence: claudia.stracquadanio@unirc.it

Tomato fruit rot is a serious disease caused by the *Fusarium* species. The aim of this study is to evaluate the ability of 2 strains of *Trichoderma* (*T. asperellum* and *T. atroviride*) to inhibit *Fusarium* spp.

The inhibitory effect of *Trichoderma* extract with ethylacetate (EtOAc) was studied in vitro and in vivo. *Trichoderma* was grown in PDB at 30 °C for 30 days. The cultured filtrate was extracted with EtOAc. The extract of *T. asperellum* (TaE) and *T. atroviride* (TatE) were tested in vitro against 6 species of *Fusarium* (in 96-well plates), obtaining the minimum inhibitory concentration and the minimum fungicidal concentration (MIC and MFC).

Starting from MFC, three concentrations were tested in vivo on tomato fruits. Tomato fruits were inoculated with *F. verticillioides*, treated with the extracts at 3 different concentrations (ranges 0.78–3.12 mg/mL), and incubated at 4 and 11 days to room temperature.

The extracts showed MIC values ranging between 0.19 and 0.78 and MFC values ranging between 0.78 and 1.56 mg/mL for TaE and TatE, respectively.

In vivo, both extracts (at 3.12 mg/mL) showed efficacy in inhibiting the growth of *F. verticillioides* with a significant difference (*p* < 0.05) compared to control (untreated). At 4 days after treatment, infection rates (IR%) of 7.5% and 23%, and Log CFU/g of 2.9 and 5.2 were for TaE and TatE, respectively. However, only TatE showed persistent efficacy at 11 days with an IR% of 19% and 4.8 Log CFU/g.

**Keywords:** antifungal activity; Trichoderma asperellum; Trichoderma atroviride; tomato fruit rot

### 5.7. Deoxynivalenol Chemical Degradation in Wheat Kernels by Application of Ammonia Vapours

Borràs-VallverdúBernat*RamosAntonio J.MarínSoniaSanchisVicenteRodríguez-BencomoJuan JoséFood Technology Department, Agrotecnio Center, University of Lleida, 25198 Lleida, Spain*Correspondence: bernatborrasvallverdu@gmail.com

Deoxynivalenol (DON) is a mycotoxin frequently found in cereals grains like wheat, barley, oats, or maize. DON is mainly produced by *Fusarium graminearum* and *Fusarium culmorum*, both of which can cause Fusarium Head Blight disease (FHB) in cereal crops. Ingestion of DON can result in chronic and acute toxic effects, the former being the most common. Chronic toxic effects comprise altered nutritional efficiency, weight loss, and anorexia, while acute effects include nausea, vomiting, and food rejection. Because of its toxicity, most countries have regulated DON maximum levels in different foods and commodities. Recommended maximum limits have also been established for animal feed. In this study, we developed a method for degrading DON on wheat kernels by exposing them to ammonia (NH_3_) vapors. Under optimal conditions (90 °C and 2 h treatment), more than 75% of the toxin was degraded in wheat kernels contaminated with 2000 μg DON/kg. No influence of the DON concentration was observed on the toxin degradation extent (concentrations of 200, 500, and 2000 μg/kg were tested). The proposed methodology for decontaminating DON in wheat kernels could be easily scaled up in the industry without the need for complex and expensive facilities. Four degradation products derived from the reaction between DON and NH_3_ were tentatively identified. Their toxicity and biological activities were in silico evaluated, and in general, lower potential negative effects were observed in comparison to the parental mycotoxin.

**Keywords:** DON; mycotoxin; chemical treatment; detoxification; degradation products

**Acknowledgments:** This work was supported by the Spanish Ministry of Economy, Industry and Competitivity through the project “Cereal sorting and processing techniques, and their impact on deoxynivalenol contamination in baby foods” (AGL2017-87755-R) (MINECO/AEI/FEDER, UE).

### 5.8. Characterization of the Antifungal Potential of Lactic Acid Bacteria Isolated from Red Grape

D’OpazoVictor*LuzCarlosLluencaFranciscoPazMaríaMañesJordiMecaGiuseppeLaboratory of Food Chemistry and Toxicology, Faculty of Pharmacy, University of Valencia, 46100 Burjassot, Valencia, Spain*Correspondence: dotavic@uv.es

Filamentous fungi infection is the principal cause of fruit and vegetal losses in the field. Chemical pesticides are the regular response to those infections. Nevertheless, the exaggerated use of these pesticides as a normal response those these contaminations brought several environmental and health problems. New and safer methods are being tested, among them, the use of microorganisms (MO) as biopreservative strategies to become an alternative to regular pesticides. In this study, the antifungal potential of 33 MO isolated from red grape was studied. First, a Gram stain was performed to identify the MO. Then, the characterization of the antifungal activity was performed using the agar diffusion method and the minimum inhibitory concentration (MIC) and minimum fungicidal concentration (MFC) method against fungi from *Aspergillus*, *Alternaria*, *Botrytis*, *Fusarium,* and *Penicillium* genera. The antifungal agent tested was a cell-free supernatant (CFS) from MRS medium fermented by the MO. Gram stain revealed that the MO was yeast and gram-positive coccus bacteria. The Agar diffusion method showed that overall, the bacterial CFS was more active against the fungi than the CFS made by yeast. The CFS fermented by UTA6 bacteria exhibited the greatest fungal inhibition. The MIC-MFC exposed similar results, CFS fermented by bacteria had higher antifungal activity, especially UTA6 reaching MIC’s from 6.3 to 50 g/L and MFC’s from 6.3 to 100 g/L. Future investigations will focus on the identification and quantification of the compounds with antifungal activity from the CFS and the possible application as a biopreservative on red grape.

**Keywords:** lactic acid bacteria; food contamination; antifungal activity; fungi

### 5.9. Toxicological Interactions between the Mycotoxin T-2 Toxin and its Modified Forms in HEPG2 Cells and Prediction of Toxicity by in Silico Approaches

TaroncherMercedes*Rodríguez-CarrascoYelkoRuizMaría JoséLaboratory of Toxicology, Faculty of Pharmacy, University of Valencia, 46100 Burjassot, Valencia, Spain*Correspondence: Mercedes.taroncher@uv.es

The T-2 toxin (T-2) is commonly metabolized to HT-2 toxin (HT-2), Neosolaniol (NEO), T2-triol, and T2-tetraol, and they can modify the toxicity of T-2. In this study, T-2 and its modified forms were evaluated by in vitro and in silico methods. The in vitro cytotoxicity individually was evaluated by MTT and Total Protein Content (PC) assays in human hepatocarcinoma (HepG2) cells. The concentrations tested were from 12.5 to 100 nM (T-2), 18.75 to 150 nM (HT-2), from 11 to 164 nM (NEO), from 164 to 2620 nM (T2-triol), and from 209 to 3350 nM (T-2 tetraol). The order of IC50 was T-2 tetraol > T-2 triol > NEO > T-2 = HT-2. The T-2 and HT-2 evidenced the highest cytotoxic effect in HepG2 cells. Cytotoxicity of binary mycotoxins combination was evaluated at ratios 1:1 (T-2 + HT-2), 1:16 (T-2 + T-2 triol and HT-2 + T-2 triol), 1:1.4 (T-2 + NEO and HT-2 + NEO), 1:2.2 (T-2 triol + T-2 tetraol), 1:11.8 (NEO + T-2 triol), 1:26.1 (NEO + T-2 tetraol), and 1:35.4 (T-2 + T-2 tetraol and HT-2 + T-2 tetraol). All binary combinations exhibited antagonistic interactions. The ADME and toxicity profile of mycotoxins were obtained by the in silico admetSAR predictive model, which determines the approaches in order to know if these mycotoxins might be taken into consideration to support a more realistic and adequate risk assessment.

**Keywords:** T-2; cytotoxicity; metabolites; interaction; in silico

**Acknowledgments:** Proyecto financiado por la Generalitat Valenciana (España) GV/2020/020 (Conselleria d’Innovacio, Universitats, Ciencia i Societat Digital, Generalitat Valenciana).

### 5.10. Development of Hanseniospora Uvarum as a New Biocontrol Agent Against Mycotoxigenic Species

MelguizoClara*Gil-SernaJéssicaVázquezCovadongaPatiñoBelénDepartment of Genetics, Physiology and Microbiology, Faculty of Biology, Complutense University of Madrid, 28040 Madrid, Spain*Correspondence: claramel@ucm.es

Mycotoxins are toxic secondary metabolites produced by some species of filamentous fungi, which represent an important threat for human and animal health and suppose large economic losses to the agri-food sector. The development of new control methods to prevent fungal growth and mycotoxin production in foodstuffs is essential, and biocontrol has been revealed as a promising alternative to conventional fungicides. In a previous work carried out in our group, we isolated a highly predominant yeast from a vineyard soil, and it was identified as *Hanseniaspora uvarum*. In this project, we tested this yeast as a potential biocontrol agent against aflatoxin- (*A. flavus* and *A. parasiticus*), ochratoxin A- (OTA) (*A. steynii* and *A. westerdijkiae*), and fumonisin- (*F. proliferatum* and *F. verticillioides*) producing fungi. CYA plates were supplemented by a final concentration of 103 cells/mL of *H. uvarum* and a spot of spores of mycotoxigenic fungi was deposited on the center of the plates. All of the fungi studied reduced their growth rate compared to the control in the presence of the potential biocontrol agent reaching inhibition percentages of 20% and 25 % in the case of *A. flavus* and *A. westerdijkiae*, respectively. Besides, mycotoxin production was evaluated by TLC in the case of OTA and aflatoxin producers and using ELISA for fumonisin-producing *Fusarium* species. In all cases, a clear reduction in OTA and aflatoxin concentration was observed in TLC plates, whereas this decrease was estimated at 50% in the case of fumonisin concentration detected in CYA plates. These initial results are promising and show the potential of *H. uvarum* as a biocontrol agent to be used in integrated control approaches to avoid mycotoxins in different crops, including those produced using organic management.

**Keywords:** biocontrol agent; fumonisins; ochratoxin A; aflatoxins; *Hanseniaspora uvarum*

**Acknowledgments:** The authors thank the AgroBank-UdL Chair “Quality and Innovation in the Agri-Food Sector” for economic aid for the realization of this work.

### 5.11. Chemical Degradation of Patulin in Apple Juices through the Formation of the Toxin-Glutathione Conjugates Induced by Pulsed Light Processing

Rodríguez-BencomoJuan José*SanchisVicenteViñasInmaculadaMartín-BellosoOlgaSoliva-FortunyRobertFood Technology Department, Agrotecnio Center, University of Lleida, 25198 Lleida, Spain*Correspondence: jrbencomo@gmail.com

Apple products are considered to be by far the most significant dietary source of patulin. Its origin is related to the contamination of the raw materials by molds (mainly by *Penicillium expansum*). In humans, the unhealthy effects of this mycotoxin include gastrointestinal disorders, nausea, and vomiting, thus that its contents in apple-based products are regulated by the food safety authorities. The removal or degradation of this mycotoxin in contaminated apple juices has been studied with different approaches with uneven effectiveness. An innovative approach for patulin degradation/detoxification could be a chemical degradation process based on the formation of specific chemical compounds. In this way, the target compounds could be those generated from the patulin cell detoxification process, namely patulin-glutathione conjugates. In this study, the reaction of patulin and glutathione (GSH) induced by pulsed light and catalyzed by ferrous ions (Fe^2+^) was evaluated in apple juice. Four processing parameters (GSH and Fe^2+^ concentrations, number of light pulses, and the thickness of the liquid phase) were studied and optimized through a central composite experimental design and a surface response analysis. In addition, the type of patulin-glutathione conjugates formed was tentatively identified. Results showed effective reductions of patulin contents (up to around 60%) with an optimum dose of GSH of 150 mg/L, the minimum thickness of liquid phase assayed, and a pulsed light energy dose of 1.2 J/cm2·mL. The catalytic effect of the ferrous ions was adequate for a molar ratio GSH/Fe^+2^ = 5. Mono-substituted patulin-glutathione adducts were identified as the main type of generated conjugates. A plus of this degradation strategy is the use of non-thermal processing of the juice to promote the chemical reaction would also allow keeping the food organoleptic/nutritional characteristics. However, more research is necessary to evaluate the stability and behavior of these adducts in the body after ingestion.

**Keywords:** patulin; apple juice; degradation; patulin-glutathione adducts; pulsed light

### 5.12. Epigenetic Changes Study in Rat Ovaries after Subchronic Oral Exposure to Enniatin A

ManyesLara^1^*LozanoManuel^1^KorreHelena^2^PinedaDaniela^2^BrobergKarin^2^1Laboratory of Food Science and Toxicology, Faculty of Pharmacy, University of Valencia, 46100 Burjassot, Valencia, Spain2Division of Occupational and Environmental Medicine, Lunds Universitet, 223 63 Lund, Sweden*Correspondence: lara.manyes@uv.es

Enniatins are secondary fungal metabolites and worldwide natural contaminants of several food and feed products. A 28-day repeated dose preliminary assay, using enniatin A naturally contaminated feed through microbial fermentation by a *Fusarium tricinctum* strain, was carried out employing 2-month-old female Wistar rats. In order to simulate a physiological test of a toxic compound naturally produced by fungi, 5 treated animals were fed for 28 days with fermented feed. As a control group, 5 rats were fed with standard feed. The estimated amount of enniatin A in serum were: 22.43, 29.02, and 36.80 μg on the 2nd, 3rd, and 4th week, respectively; and enniatin A blood concentrations obtained were: 0.97, 1.25, and 2.70 μg/mL on the 2nd, 3rd and 4th week, respectively. Previous results revealed that the relative number of lymphocytes T cytotoxic cells in the treated rats was inhibited significantly with respect to the control ones (*p* < 0.001), while lymphocytes T helper cells increased significantly (*p* < 0.001). In this study, the epigenetic alterations are evaluated in the ovaries of the treated and control rats described, in particular relative telomere length and mitochondrial DNA copy number by real-time PCR and DNA methylation by pyrosequencing. Results regarding telomere length were 1040.29 (1375.40–637.06) for enniatin A exposed rats and 1212.23 (1596.76–674.74) for control (*p* = 0.55). The mitochondrial DNA copy number showed a media of 303.73 (611.99–20.86) for treated rats and 501.97 (952.01–66.92) for control (*p* = 0.51). Results from DNA methylation by pyrosequencing are still being processed. Even if non-significant results were obtained, a decreasing tendency in mitochondrial DNA copy number and telomere length was observed, thus in the future, a larger number of samples is recommended to be analyzed in order to obtain more concluding results.

**Keywords:** epigenetics; mycotoxin; relative telomeres length; mitochondrial DNA copy number; pyrosequencing

**Acknowledgments:** Ajuda a la mobilitat de la Generalitat Valenciana (BEST/2020/161).

### 5.13. The Main Animal Source Foods Contaminated by Mycotoxins

TolosaJosefa*Vila-DonatPilarRuizMaría JoséFerrerEmiliaLaboratory of Food Chemistry and Toxicology, Faculty of Pharmacy, University of Valencia, 46100 Burjassot, Valencia, Spain*Correspondence: josefa.tolosa@uv.es

Mycotoxins are common contaminants in raw materials and feedstuffs intended for livestock. Thus, when animals are fed contaminated feeds, the mycotoxin carry-over into animal organs, edible tissues, and by-products (milk, egg, etc.) can occur. From the public health standpoint regarding mycotoxin occurrence in animal source foods (ASF), the mycotoxins considered to be of the outermost importance are aflatoxin M1 (AFM1) in milk and Ochratoxin A (OTA) in meat products, both classified as possibly carcinogenic to humans (group 2B) by the International Agency for Research on Cancer (IARC). Maximum Levels (MLs) for AFM1 in milk have been set by the European Commission (0.05 μg/kg). However, no MLs have been set in Europe for OTA in meat or meat by-products. Notwithstanding, some countries have enforced MLs of OTA concentrations, and other countries have developed national guidelines for OTA levels. The aim of this work was to review recent data reported on the AFM1 occurrence in milk and OTA in meat by-products. On the one hand, AFM1 has been widely assayed in milk samples, exhibiting differences depending on the animal species and the season of sampling, showing high contents in cow milk collected during winter season and levels reaching up to 4.2 μg/kg, exceeding the MLs, have been reported. On the other hand, regarding OTA, different surveys reported their occurrence, especially in dry-cured meat products made from pork tissues, mainly sausages, ham, and different types of salami and prosciutto. High incidence and contents have been reported ranging from 0.06 to 14.7 μg/kg, although high values (up to 691 μg/kg) have been detected in salami samples. This fact highlights the need to establish an ML for OTA in these products to protect human health and to constantly monitoring mycotoxin occurrence in animal by-products.

**Keywords:** mycotoxins; aflatoxin M1; ochratoxin A; milk; meat

**Acknowledgments:** GV/2020/020.

### 5.14. Occurrence of Mycotoxigenic Molds in Citrus Fruits of the Mediterranean Area and Biopreservative Solutions

CalpeJorge*LuzCarlosD’OpazoVictorQuilesJuan ManuelMecaGiuseppeDepartment of Preventive Medicine and Public Health, Food Sciences, Toxicology and Forensic Medicine, Faculty of Pharmacy, University of Valencia, 46100 Burjassot, Valencia, Spain*Correspondence: Jorge.calpe@uv.es

Citrus is the most important fruit crop in terms of value worldwide. In 2016, the FAO (2017) estimated the world’s citrus production at about 124.2 million tons, with orange accounting for around 67 million tons. Globally, about 27 million tons of citrus fruits are transformed industrially, mostly for the production of juice. Quality standards, the health of the consumers, and a long shelf-life are fundamental aspects affecting the competitiveness of citrus fruits produced by Mediterranean countries on both domestic and international markets. Rots caused by fungi are the main cause of post-harvest losses (estimated average of 30%) of citrus fruits and may consistently reduce their shelf life. Some fungal infections do not always cause direct damage or visible symptoms, and some citrus fungal pathogens produce toxins that could pass into the endocarp and contaminates juices too. In this study, the fungal occurrence and mycotoxin presence in citrus fruits were studied. It has been investigated in parallel the presence of lactic acid bacteria from the natural microbiota of citrus fruits as a biocontrol solution to prevent the fungal presence in peel and juice, reducing post-harvest rots and mycotoxins contents, respectively. Finally, the isolated bacteria will be tested against filamentous fungi in vitro assay to select candidates to biopreservative agents production from orange by-products and its applications like polymeric antifungal films or coatings.

**Keywords:** citrus fruits; mycotoxigenic fungi; lactic acid bacteria; by-products; biopreservation

### 5.15. Co-Occurrence of Mycotoxins in Pig Feeds from Spain

Gámiz-GraciaLaura^1^*Arroyo-ManzanaresNatalia^2^Rodríguez-EstévezVicente^3^García-CampañaAna M.^1^1Department of Analytical Chemistry, Faculty of Sciences, University of Granada, 18071 Granada, Spain2Department of Analytical Chemistry, Faculty of Chemistry, University of Murcia, 30003 Murcia, Spain3Department of Animal Production, Faculty of Veterinary, University of Cordoba, 14071 Cordoba, Spain*Correspondence: lgamiz@ugr.es

The European Commission has established regulatory levels for aflatoxin (AF) B1 and ergot sclerotia and guidelines for deoxynivalenol (DON), zearalenone (ZEA), ochratoxin A (OTA), fumonisin B1 and B2 (FB1 and FB2), T-2 and HT-2 toxins in raw materials and feed, considered as the first link in the food chain. Moreover, the European Food Safety Authority has published different scientific opinions about the risks to animal health related to the presence of some emerging mycotoxins as enniatins (ENN) and beauvericin (BEA).

In this work, 31 mycotoxins [(AF B1, AF B2, AF G1 and AF G2, OTA, FB1 and FB2, citrinin (CIT), ZEA, DON, fusarenon X (FX), sterigmatocystin (STE), T-2 and HT-2 toxin, ENN A, ENN A1, ENN B, ENN B1, BEA, ergometrine (Em), ergosine (Es), ergotamine (Et), ergocornine (Eco), ergokryptine (Ekr), ergocristine (Ecr), ergometrinine (Emn), ergosinine (Esn), ergotaminine (Etn), ergocorninine (Econ), ergokryptinine (Ekrn), and ergocristinine (Ecrn)] were determined by HPLC-FLD or UHPLC-MS/MS after solid-liquid extraction or QuEChERS. After analysis of 228 pig feed samples, the mycotoxins most frequently detected were ENN B (100% samples), ENN B1 (83.3%), ENN A1 (73.2%), BEA (98.2%), and FB1 (66.2%) while AF G2, Etn, Econ, Ecr, Ekr, and Ekrn were not detected in any sample. The highest concentrations were found for ZEA (7681 μg/kg), FB1 (3959 μg/kg), and ENN B (1222 μg/kg). Regarding co-occurrence frequency, it can be summarised as follows: 2–3 mycotoxins: 9.2%; 4 mycotoxins: 16.7%; 5 mycotoxins: 16.7%; 6 mycotoxins: 16.2%; 7 mycotoxins: 18.9%; 8 mycotoxins: 13.2%; 9–13 mycotoxins: 9.2%.

As a summary, the majority of the samples were in accordance with EU regulations (which do not address emerging mycotoxins), although 3.1% of samples showed contents of ZEA above the recommended levels. However, the high co-occurrence should be a matter of concern, as synergistic or additive effects could increase the toxicity of mycotoxins.

**Keywords:** co-occurrence; mycotoxins; feed; pig

### 5.16. Emerging Technologies to Mitigate Aflatoxin B2 in Grape Juice 

PallarésNoelia*BerradaHoudaFernández-FranzónMónicaFerrerEmiliaLaboratory of Food Chemistry and Toxicology, Faculty of Pharmacy, University of Valencia, 46100 Burjassot, Valencia, Spain*Correspondence: noelia.pallares@uv.es

Nowadays, people are seeking freshness, high vitamin content, minerals, and low-calorie products (Mandappa et al., 2018). This fact encouraged the implementation of non-thermal food processing techniques, such as high-pressure processing (HPP) and pulsed electric fields (PEF), with low impact on food nutritional components (Picart-Palmade et al., 2018). These technologies are explored for mycotoxin reduction or elimination without producing toxic residues (Gavahian et al., 2020). Aflatoxins (AFs) constituted one of the most investigated mycotoxins and were reported in food commodities such as groundnuts, sesame seeds, millet, maize, rice, wheat, fig, spices, and cocoa (Mahato et al., 2019). AFs are carcinogenic, mutagenic, and immuno-suppressive compounds that have been correlated with liver cancer (Marín et al., 2013). The European Commission (EC) has set maximal concentrations of AFB1, and the sum of AFB1, AFB2, AFG1, and AFG2 in certain foodstuffs, but maximum levels of AFs have not been set in juices (EC, 2006). The aim of this study is to investigate the effect of HPP and PEF technologies on AFB2 reduction in grape juice. Grape juice samples were spiked with AFB2 at a concentration of 100 μg/L and treated by HPP and PEF technologies. PEF treatment was carried out under conditions of the field strength of 3 Kv/cm and specific energy of 500 KJ/kg. HPP treatment conditions were set in pressure at 500 MPa for 5 minutes. After both treatments, AFB2 was extracted employing dispersive liquid-liquid microextraction method (DLLME) and determined by liquid chromatography coupled to tandem mass spectrometry (HPLC-MS/MS-IT). The reductions observed were about 72% after PEF treatment and 14% after HPP. Moreover, an AFB2 degradation product with m/z 355.0711 has been identified by quadrupole time of flight mass spectrometry detector (qTOF-MS) after PEF treatment.

**Keywords:** aflatoxin B2; HPP; PEF; decontamination; grape juice

**Acknowledgments:** The research was supported by the Spanish Ministry of Economy and Competitiveness AGL 2016-77610R and by the pre-PhD program of University of Valencia “Atracció de Talent” (UV-INV-PREDOC16F1-384781). The authors also thank Generalitat Valenciana for the financial support (IDIFEDER/2018/046 – Procesos innovadores de extracción y conservación: pulsos eléctricos y fluidos supercríticos) through European Union ERDF funds (European Regional Development Fund).

### 5.17. Predicting Toxicity and Metabolomic Profile in Silico for Zearalenone, α -Zearalenol and β -Zearalenol

AgahiFojan*JuanCristinaJuan-GarcíaAnaLaboratory of Toxicology, Faculty of Pharmacy, University of Valencia, 46100 Burjassot, Valencia, Spain*Correspondence: fojan@alumni.uv.es

Mycotoxins are present in stored grain, and co-exposure through the diet is very common. Although several studies have been done in vitro and in vivo, the toxic effects associated with metabolites generated once ingested are still unknown and difficult to study. The present study defines the metabolomics profile of all three mycotoxins (zearalenone (ZEA), α-zearalenol (α-ZEL), and β-zearalenol (β-ZEL)) and explores the prediction of their toxic effects proposing an in silico workflow by using three programs of predictions: MetaTox, SwissADME, and PASS online. Metabolomic profile was also defined and toxic effect evaluated for all metabolite products from Phase I and II reaction (a total of 15 compounds).

Results revealed that products describing metabolomics profile were: From O-glucuronidation (1z and 2z for ZEA and 1ab, 2ab, and 3ab for ZEA´s metabolites), S-sulfation (3z and 4z for ZEA and 4ab, 5ab, and 6ab for ZEA´s metabolites), and hydrolysis (5z and 7ab for ZEA´s metabolites, respectively). Lipinsky´s rule-of-five was followed by all compounds except those coming from O-glucuronidation (HBA > 10). Metabolite products had better properties to reach the blood–brain barrier than initial mycotoxins. According to Pa values (probability of activation) order of toxic effects studied was carcinogenicity > nephrotoxic > hepatotoxic > endocrine disruptor > mutagenic (AMES TEST) > genotoxic. Prediction of inhibition, induction, and substrate function on different isoforms of Cytochrome P450 (CYP1A1, CYP1A2, CYP2C9, and CYP3A4) varied for each compound analyzed; similarly, for activation of caspases 3 and 8. Relying on our findings, the metabolomics profile of ZEA, α-ZEL, and β-ZEL analyzed by in silico programs predicted alteration of systems/pathways/mechanisms that end up causing several toxic effects, giving an excellent sight and direct studies before starting in vitro or in vivo assays contributing to the 3Rs principle. However, confirmation can only be demonstrated by performing those assays.

**Keywords:** zearalenone; metabolomics; prediction; SwissADME; PASS online; MetaTox; in silico

### 5.18. Application of the Yellow Mustard Bran in Bread as a Natural Preservative Agent

TorrijosRaquel*NazarethTiago de MeloMorunoMariaMañesJordiMecaGiuseppeDepartment of Preventive Medicine, University of Valencia, 46100 Burjassot, Valencia, Spain*Correspondence: Raquel.Torrijos@uv.es

Fungal spoilage causes important economic losses in the food industry and is a challenge in food safety due to the production of mycotoxins, highly toxic compounds. In the past years, multiple strategies have been evaluated using natural compounds to prevent fungal spoilage. The aim of the study was to evaluate the antifungal properties of Yellow Mustard Seed (YMS) and Yellow Mustard Bran (YMB) extracts against toxigenic fungi of the *Aspergillus*, *Penicillium* and *Fusarium* genera. For this, a qualitative evaluation test on PDA plates was performed. YMB evidenced the highest antifungal activity, and, for this reason, the Minimum Inhibitory Concentration (MIC) and Minimum Fungicidal Concentration (MFC) were established in vitro. The *Penicillium* genera evidenced the lowest MIC and MFC values, ranging from 0.3 to 4.7 g/L. Then, the use of YMB was studied to increase the shelf life of bread contaminated with *P. commune* CECT 20767. For this, different amounts of YMB were tested (2.5, 5, 7.5, and 10 g/Kg). In addition, a commercial treatment with sodium propionate (E-281) and a control treatment without preservatives was performed. The use of 10 g/Kg increased the shelf life in comparison to the control by 3 days and, in addition, equated the reduction of the fungal population as the commercial treatment. These results suggest the promising employment of YMB as an antifungal additive in the food industry because they satisfy the consumers’ demand for natural additives.

**Keywords:** yellow mustard; antifungal activity; *Penicillium*; bread

### 5.19. Occurrence of Aflatoxin M1 in Ovine Milk from Spanish Dairy Sheep Herds

BodasRaúl^1^CarbajoRaúl^1^JuanTeresa^2^,^3^*HerreraMarta^3^IguacelLaura^2^LoránSusana^3^CarramiñanaJuan José^3^YagüeCristina^4^GiráldezFrancisco Javier^5^García-GarcíaJuan José^1^AriñoAgustín^3^1Instituto Tecnológico Agrario de Castilla y León, Subdirección de Investigación y Tecnología, Área de Investigación Ganadera, Línea de Rumiantes, 47071 Valladolid, Spain2Centro de Investigación y Tecnología Agroalimentaria de Aragón (CITA), 50059 Zaragoza, Spain3Instituto Agroalimentario de Aragón-IA2 (CITA-Universidad de Zaragoza), Veterinary Faculty, University of Zaragoza-CITA, 50013 Zaragoza, Spain4Faculty of Health and Sports Science, 22002 Huesca, Spain5Instituto de Ganadería de Montaña (CSIC-Universidad de León), Grulleros, 24346 León, Spain*Correspondence: tjuan@cita-aragon.es

The presence of aflatoxin M1 (AFM1) in sheep milk is becoming a concern for farmers, industry, and consumers. In order to examine the presence of AFM1 in ovine milk, bulk tank milk samples were collected from 51 dairy sheep farms from Castilla and León (Spain) in autumn 2019. Each farmer was interviewed and a questionnaire was filled to characterize farm management and productivity at the time of sample collection: Number of total and milking ewes, daily milk production, type of milking parlour, number of farm workers, bedding frequency, use of total mixed rations, use of adsorbents, and previous mycotoxicoses. Aflatoxin M1 was determined by two methods: (a) A rapid test (lateral flow immunoassay, LFI) with a limit of detection (LOD) of 8 ng/L and (b) ultra-high-performance liquid chromatography with a fluorescence detector (UPLC-FLD) with a LOD of 0.92 ng/L. Samples below LOD were considered as negative and assigned a value of zero. Both methods detected a different number of positive samples; thus, 37% of samples were positive by LFI and 88% by UPLC-FLD. The average values observed in the positive samples were 11.2 and 3.8 ng AFM1/L milk for LFI and UPLC-FLD, respectively. The preliminary analyses did not allow to link the occurrence of AFM1 in milk with any of the studied farm characteristics. Therefore, albeit AFM1 could be found in ovine milk, it must be highlighted that the observed concentrations were far below the EU maximum level (50 ng/L).

**Keywords:** aflatoxin M1; milk; dairy; sheep; farm

**Acknowledgments:** The authors thank the financial support from the Ministry of Science and Innovation (INIA RTA 2017-00085-C2) and the Government of Aragón and FEDER 2014-2020 (Grant Grupo A06_20R).

### 5.20. Aflatoxin Carry-Over Rates in Assaf Lactating Ewes

JuanTeresa^1^,^2^*BodasRaúl^3^HerreraMarta^2^IguacelLaura^1^BertolínJuan Ramón^1^,^2^CarbajoRaúl^3^LoránSusana^2^CarramiñanaJuan José^2^YagüeCristina^4^GiráldezFrancisco Javier^5^LópezSecundino^5^AriñoAgustín^2^1Centro de Investigación y Tecnología Agroalimentaria de Aragón (CITA), 50059 Zaragoza, Spain2Instituto Agroalimentario de Aragón-IA2 (CITA-Universidad de Zaragoza), Veterinary Faculty, University of Zaragoza-CITA, 50013 Zaragoza, Spain3Instituto Tecnológico Agrario de Castilla y León. Subdirección de Investigación y Tecnología, Área de Investigación Ganadera, Línea de Rumiantes, 47071 Valladolid, Spain4Faculty of Health and Sports Science, 22002 Huesca, Spain5Instituto de Ganadería de Montaña (CSIC-Universidad de León), Grulleros, 24346 León, Spain*Correspondence: tjuan@cita-aragon.es

Aflatoxins are mycotoxins contaminating raw materials, feed and food, and known for their genotoxic and carcinogenic effects on various animal species and humans (Group 1 by IARC). Raw milk is contaminated with the hydroxy-metabolite aflatoxin M1, following exposure of lactating animals to aflatoxin B1 present in feedstuffs. Most studies on the carry-over of aflatoxins present in feedstuff into milk have been reported for cows, whereas research on lactating ewes is very scarce despite the great importance of the latter in the Mediterranean area. This study aimed to investigate the aflatoxin carry-over in dairy sheep. Thirty Assaf ewes in mid-lactation, individually penned, fed, and milked were divided into 3 experimental groups, each animal receiving a different daily dose of AFB1 over 13 days: 0 (Control); 40 μg (L) and 80 μg (H). Milk samples were collected at 0, 1, 2, 3, 6, 13, 14, 16, and 17 days. After IAC cleanup, AFM1 was analyzed by UPLC coupled to fluorescence detector, with a limit of detection of 0.92 ng/L.

AFM1 was detected in both L and H groups from day 1 to 14, AFM1 concentration in group H (69 ng/L) exceeding the EU maximum level (50 ng/L) on day 1. AFM1 excretion pattern was similar in L and H groups, with an increase until days 3–5, and then a gradual decline until reaching a stable concentration. AFM1 was not detected in milk from the 2nd day after removal of the contaminated feed. The carry-over rate of aflatoxins from feed to milk was nearly 0.2% on average (lower than the 1–2% reported for cows). It was found that the carry-over rate was higher in L group than in H group (*p* < 0.05).

**Keywords:** aflatoxins; dairy sheep; carry-over; feed; milk

**Acknowledgments:** The authors thank the financial support of the Ministry of Science and Innovation (INIA RTA 2017-00085-C2) and the Government of Aragón and FEDER 2014-2020 (Grant Grupo A06_20R).

### 5.21. Occurrence of Aflatoxins in Cocoa Powder Samples Marketed in Spain

HerreraMarta^1^*SalasGuillermo^1^LoránSusana^1^JuanTeresa^1^CarramiñanaJuan José^1^YagüeCristina^2^HerreraAntonio^1^AriñoAgustín^1^1Instituto Agroalimentario de Aragón-IA2, Veterinary Faculty, University of Zaragoza-CITA, 50013 Zaragoza, Spain2Faculty of Health and Sports Science, University of Zaragoza, 22002 Huesca, Spain*Correspondence: herremar@unizar.es

Aflatoxins pose a serious risk to food safety, contaminating crops in the field and during storage and affecting a wide variety of raw materials and processed foods. In the recent EFSA risk assessment of aflatoxins in foods, cocoa and cocoa products made one of the largest contributions to the dietary exposure to aflatoxin B1 in all age classes. Thus, it is important to investigate their occurrence in these products that indeed are usually consumed by vulnerable population groups such as children. In addition, there is currently a certain legal gap in EU legislation since the maximum level for aflatoxins, which are carcinogenic to humans, is not regulated for cocoa and derived products.

The aim of this study was to evaluate the occurrence of aflatoxins B1, B2, G1, and G2 in 71 commercial samples of branded cocoa powder (13 organic and 58 conventional). Mycotoxins were extracted with methanol:water (80:20) followed by cleanup using immunoaffinity columns. Finally, the determination was made by HPLC coupled to photochemical (PHRED) and fluorescence (FLD) detectors, with a limit of detection of 0.02 μg/kg for each of the aflatoxins. There were 37 out of 71 samples positive for total aflatoxins, with levels ranging from 0.02 μg/kg to 3.33 μg/kg. The presence of the different aflatoxins was: B1 (27 samples), G1 (15 samples) and B2 (9 samples); no aflatoxin G2 was detected. Aflatoxins B1, B2, and G1 were detected simultaneously in four samples, B1 and G1 in six samples, while B1 and B2 coexisted in eight samples. The incidence of total aflatoxins was similar in organic (46%) and conventional (53%) cocoa samples. The appropriateness of setting a maximum level for aflatoxins in cocoa and derived products should be considered in the light of public health risks.

**Keywords:** aflatoxins; cocoa; children; HPLC

**Acknowledgments:** The authors thank the financial support of the Ministry of Science and Innovation (PID2019-106877RA-I00) and the Government of Aragón and FEDER 2014-2020 (Grant Grupo A06_20R).

### 5.22. Cytotoxicity of Mycotoxins in the RTgill-W1 Fish Cell Line

BernalElenaPulgarínMartaFernández-CruzMaría Luisa*Department of Environment and Agronomy, INIA-National Institute for Agricultural and Food Research and Technology, 28040 Madrid, Spain*Correspondence: fcruz@inia.es

In the last decades, the demand for fish as a protein source for human consumption has increased, leading to some adaptations in the aquaculture industry. One of the most important changes is the introduction of vegetables as a source of protein in feeds, giving rise to new concerns about its contamination with mycotoxins. Mycotoxins in aquafeeds can represent an ecological, health, and economic problem, and it is necessary to for better knowledge about the occurrence of mycotoxins in aquafeeds and their toxicity or bioaccumulation in fish.

The objective of this study was to screen in vitro the toxicity of 15 mycotoxins to the fish. The RTgill-W1 fish cell line was exposed to a range of mycotoxins concentrations (0.012-100 µg/mL) during 24 hours. The cytotoxicity was evaluated with triple assay (AlamarBlue, CFDA-AM, and Neutral Red Uptake), which measures the cell metabolism at the mitochondrial level and the plasma lysosome membrane integrities, respectively.

Mycotoxins were toxic mainly at the lysosomal level with IC50 values of 2.82 ± 0.41, 2.88 ± 0.57, 3.98 ± 0.97, 6.04 ± 0.84, 8.03 ± 1.71, 23.50 ± 2.19, 43.51 ± 1.71, 53.03 ± 11.01, and 81.44 ± 11.92 µg/mL for Beauvericine, Enniantin A, Enniantin A1, Enniantin B1, Enniantin B, Zearalenone, 15-O-ac-Deoxynivalenol, Ochratoxine, and 3-ac-Deoxynivalenol, respectively. Aflatoxins (AF) exerted the highest effect at the mitochondrial level with IC50 of 11.45 ± 3.47 and 25.90 ± 7.65 for AFB1 and AFB2, respectively. In general, the plasma membrane was less affected by the mycotoxins. Fumonisins (FB1, FB2), Nivalenol, and Deoxynivalenol were the less toxic with IC50 > 100 µg/mL.

The results indicate that most of the mycotoxins assayed exert a high acute effect in the fish cell line, indicating a possible concern for the aquaculture industry and for the consumers. Further research is needed to study the toxic and bioaccumulation profile of these mycotoxins in the fish.

**Keywords:** mycotoxin; cytotoxicity; viability; RTgill-W1; fish

### 5.23. Study of Mycotoxins in Maize Consumed in Spain by an Optimized UPLC–(ESI+)–MS/MS Method

GómezJosé Vicente^1^TarazonaAndrea^1^MateoFernando^2^Gimeno-AdelantadoJosé Vicente^3^JiménezMisericordia^1^García-EsparzaMaría Ángeles^4^SoriaJosé Miguel^5^MateoEva María^6^*1Department of Microbiology and Ecology, University of Valencia, 46100 Burjassot, Valencia, Spain2Department of Electronic Engineering, ETSE, University of Valencia, 46100 Burjassot, Valencia, Spain3Department of Analytical Chemistry University of Valencia, 46100 Valencia, Spain4Department of Pharmacy, School of Health Sciences, University Cardenal Herrera CEU, 03204 Elche, Alicante, Spain5Department of Biomedical Sciences, School of Health Sciences, Universidad Cardenal Herrera-CEU, 03204 Elche, Alicante, Spain6Department of Microbiology, School of Medicine, University of Valencia, 46010 Valencia, Spain*Correspondence: Eva.mateo@uv.es

The occurrence of mycotoxins in maize kernels (*Zea mays* L.) is of great concern worldwide and is often associated with mycotoxicosis in livestock and in humans. In Spain, maize is produced all over the country but mainly in the regions Castile and Leon, Aragon, and Extremadura, followed by Catalonia, Castile-La Mancha, Navarre, Andalusia, and Galicia. The most prominent mycotoxins in cereals are aflatoxins B1, B2, G1, and G2 (AFB1, AFB2, AFG1, and AFG2), fumonisins B1 and B2 (FB1 and FB2), ochratoxin A (OTA), zearalenone (ZEA), deoxynivalenol (DON), 3- and 15-acetyl-deoxynivalenol (3- and 15-ADON), and T-2 and HT-2 toxins. The goal of the present study was to investigate the occurrence of these mycotoxins in maize kernels commercialized and consumed in Spain. For this purpose, a sensitive, rapid, and reliable UPLC–(ESI+)–MS/MS multi-mycotoxin method using matrix-matched calibration for the determination of target mycotoxins in maize kernels was validated and applied to study the distribution of these mycotoxins in 98 maize kernels samples collected in 26 stores across different Spanish regions for a 5-year period (2015–2019). FB1, FB2, DON, 3-ADON, ZEA, AFB1, AFB2, AFG2, T-2 and HT-2 were quantified in 71.4%, 56.1%, 31.6%, 5.1%, 24.5%, 9.2%, 9.2%, 2%, 5.1%, and 5.1% of samples, respectively. The maximum EU limits for FB1 + FB2, ZEA, AFB1, and the sum of aflatoxins, were exceeded in 20.4%, 5.1%, 3%, and 3% of the analyzed samples, respectively. OTA and AFG1 were not detected, and the sum of T-2 + HT-2 was below the EU recommended limit. Various mycotoxins (two to seven) co-occurred at levels ≥ LOQ in about 34% of the samples, which may increase toxicity due to possible additive or synergistic effects. The results obtained in this study contribute to increase the knowledge on mycotoxin contamination of maize and warn about the need to assess mycotoxin levels in food and feed derived from corn.

**Keywords:** maize; mycotoxins; UPLC–(ESI+)–MS/MS method; co-occurrence; fumonisins

**Acknowledgments:** The authors acknowledge financial support from the Ministry of Economy and Competitiveness (MINECO, Spanish Government) (Project RTI2018-097593-B-C22).

### 5.24. Prediction of F. sporotrichioides Growth and T-2 and HT-2 Accumulation in Oats in the Presence of EVOH-Essential Oil Films Using Machine Learning Methods

MateoEva María^1^GómezJosé Vicente^2^TarazonaAndrea^2^GavaraRafael^3^MateoFernando^4^*1Department of Microbiology, School of Medicine, 46010 Valencia, Spain2Department of Microbiology and Ecology, University of Valencia, 46100 Burjassot, Valencia, Spain3Institute of Agrochemistry and Food Technology (CSIC), 46980 Paterna, Valencia, Spain4Department of Electronic Engineering, ETSE, University of Valencia, 46100 Burjassot, Valencia, Spain*Correspondence: Fernando.mateo@uv.es

Toxigenic fungi have very negative consequences on human society. These fungi and their mycotoxins cause devastating effects on agricultural crops, the economy, food security, and human and animal health. T-2 and HT-2 toxins (T-2 and HT-2) are considered two of the most relevant mycotoxins in cereals in Europe, especially in oats. In Southern countries with warmer weather, the species *Fusarium sporotrichioides* is considered the most relevant species concerning T-2 and HT-2 production in cereals. These mycotoxins inhibit DNA, RNA, and protein synthesis and induce DNA fragmentation characteristic of apoptosis. There are currently various approaches to reduce toxigenic fungi and mycotoxins, but the problems associated with T-2 and HT-2 in cereals in pre- and post-harvest have not been resolved. The development of active antifungal films containing pure components of essential oils (EOC) (GRAS compounds) is of great interest in food microbiology and technology. Likewise, predictive models based on machine learning (ML) algorithms might be innovative tools for the appropriate management of toxigenic fungi and mycotoxins in food. The aims of this study were: (a) To evaluate the potential of ethylene-vinyl alcohol copolymer (EVOH) containing EOC in the control of *F. sporotrichioides* growth in oats under different environmental conditions and in the control of T-2 and HT-2 production, and (b) to assess the ability of various ML methods to predict the growth rates of this fungus and T-2/HT-2 accumulation under the assay conditions.

Mycotoxins were determined by UPLC-MS/MS. Effective doses of EVOH films containing cinnamaldehyde (CINHO), isoeugenol (IEG), citral (CIT), or linalool (LIN) were calculated. Four ML methods (neural networks (NN), random forest (RF), support vector machines (SVM), and extreme gradient boosted trees (XGBoost) models were evaluated for the first time for modeling growth and T-2 and HT-2 production by *F. sporotrichioides*. The most effective films were EVOH-CIT, EVOH-IEG, and EVOH-CINHO and XGBoost and RF provided the best performance as predictive models.

**Keywords:** T-2 and HT-2 toxins; physicochemical conditions; *F. sporotrichioides*; bioactive films machine learning

**Acknowledgments:** The authors acknowledge financial support from the Ministry of Economy and Competitiveness (MINECO, Spanish Government, Project RTI2018-097593-B-C22).

### 5.25. Occurrence of Mycotoxins in Oats Consumed in Spain

TarazonaAndrea^1^GómezJosé Vicente^1^MateoFernando^2^García-DíazMarta^3^JiménezMisericordia^1^MateoEva María^4^*1Department of Microbiology and Ecology, University of Valencia, 46100 Burjassot, Valencia, Spain2Department of Electronic Engineering, ETSE, University of Valencia, 46100 Burjassot, Valencia, Spain3Department of Genetics, Physiology and Microbiology, Faculty of Biology, Complutense University of Madrid, 28040 Madrid, Spain4Department of Microbiology, School of Medicine, University of Valencia, 46010 Valencia, Spain*Correspondence: Eva.mateo@uv.es

Oats (*Avena sativa*) is a cereal belonging to the *Poaceae* grass family of plants. Although traditionally used for animal feed, oat products’ beneficial nutritional and physiological effects have generated an increased demand for oats in human nutrition. Oats have good taste, dietetic properties, high beta-glucan content, and anticarcinogenic effects. Beta-glucan is known as a prebiotic, stimulating the growth of some beneficial residential colon microorganisms, such as bifidobacteria. However, oats can be contaminated by toxigenic fungi and their associated mycotoxins in the field or during storage. The European Commission (EC) has established Maximum Levels (MLs) in oats for a selected number of mycotoxins. They are aflatoxin B1 (AFB1), the sum of aflatoxins (AFB1 + AFB2 + AFG + AFG2), deoxynivalenol (DON), zearalenone (ZEA), and ochratoxin A (OTA). MLs for the sum of T-2 and HT-2 toxins (T-2 and HT-2) in oats and other cereals have been recommended by the EC. Several LC-MS/MS methods have been reported for the determination of mycotoxins in cereals and cereal-based products, mostly for rice, maize, wheat, and barley. However, only a limited number of reports have focused on the occurrence of the mycotoxins in oats. The present study was aimed at investigating the occurrence of these and other mycotoxins in oats collected and commercialized in different Spanish regions for a five-year period (2015–2019).

One hundred samples of oat grains were collected in stores located throughout the country and analyzed for mycotoxins using a validated multi-mycotoxin UPLC-MS/MS method. AFB1, AFB2, AFG2, DON, 3-acetyl–DON, ZEA, T-2, HT-2, fumonisin B1, fumonisin B2, and OTA were detected, and their respective quantification limits were exceeded in some samples. The most frequently detected mycotoxins were ZEA, HT-2, and DON. The MLs set or recommended by the EC were surpassed in some samples by ZEA, the sum of aflatoxins, or the sum of T-2 and HT-2. Various mycotoxins co-occurred in the same sample.

**Keywords:** oats; mycotoxins; UPLC–MS/MS method; co-occurrence

**Acknowledgments:** The authors acknowledge financial support from the Ministry of Economy and Competitiveness (Spanish Government) (Project RTI2018-097593-B-C22).

### 5.26. Application of Machine Learning Methods in the Prediction of the Efficacy of Different Commercial Antifungal Formulations against Fusarium spp.

MateoFernando^1^*TarazonaAndrea^2^GómezJosé Vicente^2^SoriaJosé Miguel^3^García-EsparzaMaría Ángeles^4^MateoEva María^5^1Department of Electronic Engineering, University of Valencia, 46100 Burjassot, Valencia, Spain2Department of Microbiology and Ecology, University of Valencia, 46100 Burjassot, Valencia, Spain3Department of Biomedical Sciences, School of Health Sciences, University Cardenal Herrera-CEU, 03204 Elche, Alicante, Spain4Department of Pharmacy, School of Health Sciences, University Cardenal Herrera CEU, 03204 Elche, Alicante, Spain5Department of Microbiology and Ecology, School of Medicine, University of Valencia, 46010 Valencia, Spain*Correspondence: Fernando.mateo@uv.es

*Fusarium culmorum* is the primary etiological agent of *Fusarium* crown rot and *Fusarium* head blight, two of the most economically destructive diseases affecting cereal production worldwide. *Fusarium proliferatum* is a common pathogen infecting cereals. In addition, they are one of the main zearalenone (ZEA) and fumonisin (FB) producing species, respectively. ZEA and FB production are subjected to both genetic and physicochemical controls (water activity, temperature, fungicide treatments). Knowledge of the effectiveness of commercial antifungal formulations used in agriculture to control toxigenic species and mycotoxin production is very scarce. Particularly, there are no data about three commercial formulations: Microthiol^®^ (sulfur, 80 % WG), Escolta^®^ (trifloxystrobin, 375 g/L + cyproconazole, 160 g/L), and Raxil plus^®^ or (Lamardor^®^ FS 400) (prothioconazole, 250 g/L + tebuconazole, 150 g/L) against toxigenic fungi and mycotoxin biosynthesis. Machine learning (ML) is a multidisciplinary subject involving many disciplines, such as probability theory, statistics, approximation theory, etc. Supervised.ML can be used in prediction analysis. The goals of this study were: (1) To evaluate the effectiveness of Microthiol^®^, Escolta^®^ and Raxil plus^®^ against *F. culmorum* and *F. proliferatum* under different environmental conditions in vitro; (2) to determine the effect of these treatments on ZEA production by *F. culmorum* and on FB1 and FB2 production by *F. proliferatum* under such conditions, and (3) to design ML methods able to predict the growth rates of these fungi and mycotoxin production.

The most effective treatment was Raxil plus^®^. Its effective doses for reducing the growth to 50% (ED_50_), 90% (ED_90_) or total growth inhibition (ED_100_) ranged as follows: ED_50_ 0.49–1.70 mg/mL, ED_90_ 2.57–6.02 mg/mL, and ED_100_ 4.0–8.0 mg/mL, depending on the species, water activity, and temperature. The extreme gradient boosted tree was the ML model able to predict growth rate and mycotoxin production with minimum error and maximum R^2^ value.

**Keywords:***F. culmorum*; *F. proliferatum*; zearalenone; fumonisins; machine learning

**Acknowledgments:** The authors acknowledge financial support from the Ministry of Economy and Competitiveness (MINECO, Spanish Government) (Project RTI2018-097593-B-C22).

### 5.27. Screening of Lactic Acid Bacteria with Antifungal Activity Isolated from Dry-Cured Meats

NazarethTiago de Melo*SalcedoOscarTorrijosRaquelMañesJordiMecaGiuseppeLaboratory of Food Chemistry and Toxicology, Faculty of Pharmacy, University of Valencia, 46100 Burjassot, Valencia, Spain*Correspondence: tiago@uv.es

Fungal spoilage is not only a global food quality concern but also presents serious health problems due to the production of mycotoxins, some of which present considerable challenges to food safety and generate large economic losses. Chemical preservatives are successful in retarding microbial growth, yet the growing demand for clean label products requires manufacturers to find natural alternatives to replace chemical ingredients. Lactic acid bacteria (LAB) are generally recognized as safe (GRAS), therefore, they are considered a good candidate for their use as a natural preservative in food to control fungal growth and subsequent mycotoxin production, as well as to improve shelf life. The objectives of this study were to identify and characterize LAB isolated from dry-cured meats with the potential to inhibit the growth of five toxigenic fungi (*Aspergillus flavus* ITEM 8111, *Cladosporium oxysporum* CECT 20421, *Penicillium nordicum* CECT 2320, *Penicillium verrucosum* VTT 47, and *Penicillium griseofulvum* CECT 2605). The A. flavus proved to be more resistant against all the LAB strains tested. From a total of 90 LAB strains isolated, 7 were selected for their high growth inhibitory effect in direct contact with the fungus by overlay technique, and another 7 were selected for their capacity to inhibit fungal growth by halo diffusion assay. In addition, the ability of the bacteria to hydrolyze meat proteins was also considered. For further studies, we propose the application of fermented swine loin extracts produced by these bacteria in casings to manufacture dry-cured meat products.

**Keywords:** lactic acid bacteria; mycotoxins; dry-cured meat; antifungal; fungal spoilage

## 6. Roundtable: “Importance of Food Safety in the Post COVID 19 Industry”

### 6.1. Food Packaging Challenge in the Age of COVID-19

RodríguezLorenaAIMPLAS, The Plastic Technology Center, Technology Park, 46980 Paterna, Valencia, Spain; lrodriguez@aimplas.es

The food packaging industry is facing a series of challenges as consumers and the rest of the world deal with changes related to COVID-19.

Customer demand has shifted drastically—the pandemic shut down restaurants and food-service outlets. Consumers have moved to buy grocery purchases, for which the use of packaging has risen.

Consumers’ wishes to stockpile and their panic purchases of food, beverages, and home-care necessities have accentuated this trend.

Single-use, disposable food packaging appears to have made a comeback rising on the coattails of the COVID-19 pandemic, as many consumers believe this to be safer and/or more hygienic, but is this true?

In the food packaging challenges in the age of COVID 19 presentation, we will review, which the food packaging challenge are and how the sector can solve them.

**Keywords:** food packaging; active; edible

### 6.2. COVID 19: It Is Not a Food Crisis, But It is an Important Challenge for The Food Industry

OrtuñoRobertoAINIA Technology Centre, Technology Park, 46980 Paterna, Valencia, Spain; rortuno@ainia.es

COVID-19 is not considered a food transmission disease. The European Food Safety Agency (EFSA) is closely monitoring the information generated in the context of this crisis and has reported that there is no evidence that food is a COVID-19 source or route. The same view has been expressed by the FDA (Food and Drug Administration of the United States) and WHO.

However, the food sector has a triple challenge in this pandemic, meeting unusual demand, safeguarding worker’s health, and ensuring food safety of products. To achieve this triple objective (focusing on measures that impact food safety and worker health), it is important to strengthen hygiene practices in processing and handling operations. The food sector currently has robust food safety management systems, like APPCC (hazard analysis and critical control points), including the adoption of correct hygiene practices in mandatory form or BRC, IFS, and ISO 22.000 as non-mandatory systems.

Therefore, we can ensure that the food sector has the necessary tools to ensure food safety, but it is important to take measures to strengthen food hygiene practices and monitoring.

In the first weeks of the pandemic, we considered that it could be useful to develop the “COVID Manual. Strengthening food hygiene measures in the productive environment” in order to provide food industries with guidelines to deal with this special situation.

This manual includes measures concerning plant staff, visits, and other operations, such as maintenance, cleaning, and disinfection, etc. It has received more than 3000 downloads and has been presented in 2 webinars with more than 1000 attendees.

### 6.3. Mycotoxin Risk Management in the Food Industry

DevesaAmparoImportaco, S.A.U, 46469 Beniparrell, Valencia, Spain; adevesa@importaco.com

Nuts are suitable substrates for fungal growth if production and trade conditions are appropriate. It is well-known that some of these molds can be mycotoxigenic, thus several mycotoxins may be present in our products. According to the Food Agriculture Organization (FAO), around 25% of crops are affected by mycotoxigenic moulds worldwide and, it has been estimated that over 1000 million tons are lost each year due to this reason. Besides, according to the annual report of the Rapid Alert System for Food and Feed, nuts were two of the most affected food categories by mycotoxins. Aflatoxins were the primary mycotoxins associated with the notifications, but more and more, other types and emergent mycotoxins are also of relative concern for the food industry. Therefore, this is one of the main concerns in our enterprise.

Traditionally, food safety control in the food industry consisted in determining the levels of mycotoxins in acquired goods according to legislated levels. This is a one-side control mechanism that does not provide complete information considering the heterogeneous distribution of mycotoxins. However, in our enterprise, we designed a 360° model based on a multifactorial point of view. This model is intended to ensure food safety and quality control throughout the entire food production chain, namely from farm to fork. This way, we are capable of gathering data from several key points that enable us to work in a preventive approach instead of a corrective mode. Our final aim is to achieve a predictive model that guarantees no presence of mycotoxins in our goods from the first beginning of the chain.

**Keywords:** mycotoxin; nuts; predictive quality; multifactorial model

